# Electrothermal Transformations within Graphene-Based Aerogels through High-Temperature Flash Joule Heating

**DOI:** 10.1021/jacs.3c06349

**Published:** 2023-12-30

**Authors:** Dong Xia, Jamie Mannering, Peng Huang, Yifei Xu, Qun Li, Heng Li, Yi Qin, Alexander N. Kulak, Robert Menzel

**Affiliations:** † School of Chemistry, University of Leeds, Leeds LS2 9JT, U.K.; ‡ Department of Materials, University of Manchester, Manchester M13 9PL, U.K.; § State Key Laboratory of Molecular Engineering of Polymers, Department of Macromolecular Science, Fudan University, Shanghai 200438, China; ∥ School of Chemistry and Chemical Engineering, Chongqing University, Chongqing 400044, China; ⊥ Key Laboratory of Estuarine Ecological Security and Environmental Health, Tan Kah Kee College, Xiamen University, Zhangzhou 363105, China; # Department of Engineering Science, University of Oxford, Oxford OX1 3PJ, U.K.

## Abstract

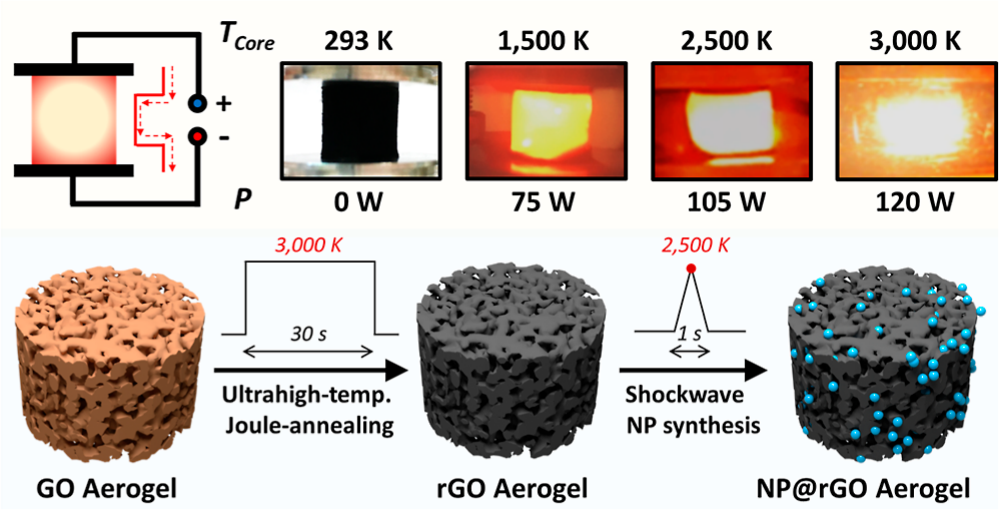

Flash Joule heating of highly porous graphene oxide (GO) aerogel monoliths to ultrahigh temperatures is exploited as a low carbon footprint technology to engineer functional aerogel materials. Aerogel Joule heating to up to 3000 K is demonstrated for the first time, with fast heating kinetics (∼300 K·min^–1^), enabling rapid and energy-efficient flash heating treatments. The wide applicability of ultrahigh-temperature flash Joule heating is exploited in a range of material fabrication challenges. Ultrahigh-temperature Joule heating is used for rapid graphitic annealing of hydrothermal GO aerogels at fast time scales (30–300 s) and substantially reduced energy costs. Flash aerogel heating to ultrahigh temperatures is exploited for the in situ synthesis of ultrafine nanoparticles (Pt, Cu, and MoO_2_) embedded within the hybrid aerogel structure. The shockwave heating approach enables high through-volume uniformity of the formed nanoparticles, while nanoparticle size can be readily tuned through controlling Joule-heating durations between 1 and 10 s. As such, the ultrahigh-temperature Joule-heating approach introduced here has important implications for a wide variety of applications for graphene-based aerogels, including 3D thermoelectric materials, extreme temperature sensors, and aerogel catalysts in flow (electro)chemistry.

## Introduction

Thermochemical transformations via the process of direct electrical heating (Joule heating) has recently come into focus as a powerful tool to control the physio-chemical properties of conductive carbon-based functional materials.^1^ Joule heating can, in principle, enable extremely fast heating kinetics (>100 K·s^–1^) and high target temperatures (>2000 K), while the Joule-heating technique itself offers a large degree of flexibility as it can be readily adapted to different material forms and chemical environments.^2−4^ High-temperature Joule-heating treatments have recently been explored in a range of carbon-based functional material systems, including for the redispersion of nanoparticles on carbon nanofiber filaments,^5^ programmable heating of carbon papers for catalyst-free CH_4_ pyrolysis,^6^ formation of high-entropy mixed metal alloys on carbon fiber filaments,^7^ phase-controlled synthesis of metal carbide catalyst on carbon black,^8^ synthesis of single-atom catalysts on electro-spun carbon nanofibers,^9^ and even the mass production of graphene itself from waste material.^10−12^ However, high-temperature Joule heating is applied almost exclusively to thin conducting carbon films^13^ or compacted powders,^14^ while the composition and structure of the conducting substrate are rarely considered in the development of high-performance materials.

Alternatively, carbon-based aerogel and foam materials provide an exciting opportunity to utilize Joule heating to control chemical transformations within complex 3D architectures opening up an opportunity to explore unique structure–function relationships within a broad range of applications from energy storage and generation to (electro)chemical catalysis and environmental remediation.^15−18^ The assembly of low-dimensional nanocarbon building blocks (graphene derivatives, carbon nanotubes) into porous aerogels has been successfully used to exploit the unique properties of nanocarbons at macroscopic scales.^19,20^ In this work, we focus on aerogels constructed from graphene oxide (GO) as a model system where a diverse range of 3D internal architectures (e.g., lamellar, macrocellular, and hierarchical porosity) have been successfully demonstrated in the literature.^21−23^ Such GO-derived aerogel materials have found a growing range of applications, e.g., as electrodes (energy storage, sensors),^24,25^ actuators,^26^ catalysts^27^ and adsorbents (water treatment),^28^ based on their combination of high surface area, tailored porosity, and excellent electro-thermal conductivity.^29^

Many practical applications of GO aerogels require thermochemical GO modification to create fully functional materials.^30^ Most typically, the GO parent material needs to be converted into reduced GO (rGO) to restore key graphitic material characteristics (especially electrical and thermal conductivity), to remove synthetic impurities, and to promote chemical cross-linking of the 3D GO network.^31^ GO can be converted into rGO via chemical reduction agents at relatively low temperatures;^32^ however, chemical reduction typically results in defect-rich rGO with comparatively low conductivities and limited mechanical properties. To promote the formation of high-quality rGO aerogels, high-temperature treatments under inert or reducing atmospheres are required. Thermochemical treatments are typically carried out through external heating processes, e.g., via high-temperature furnaces.^33^ Such furnace treatments are also often used for aerogel functionalization with functional nanoparticles, i.e., for the thermochemical conversion of aerogel-embedded molecular precursors into functional nanoparticles.^34,35^ The resulting nanoparticle-decorated rGO aerogels have shown great promise in a wide range of applications, such as batteries, supercapacitors, and sensors.^36−38^ However, furnace-based thermochemical treatments can be lengthy and inhomogeneous. Moreover, high-temperature furnaces (temperature >2000 K) are not easily available and are extremely energy-intensive.

**Scheme 1:**
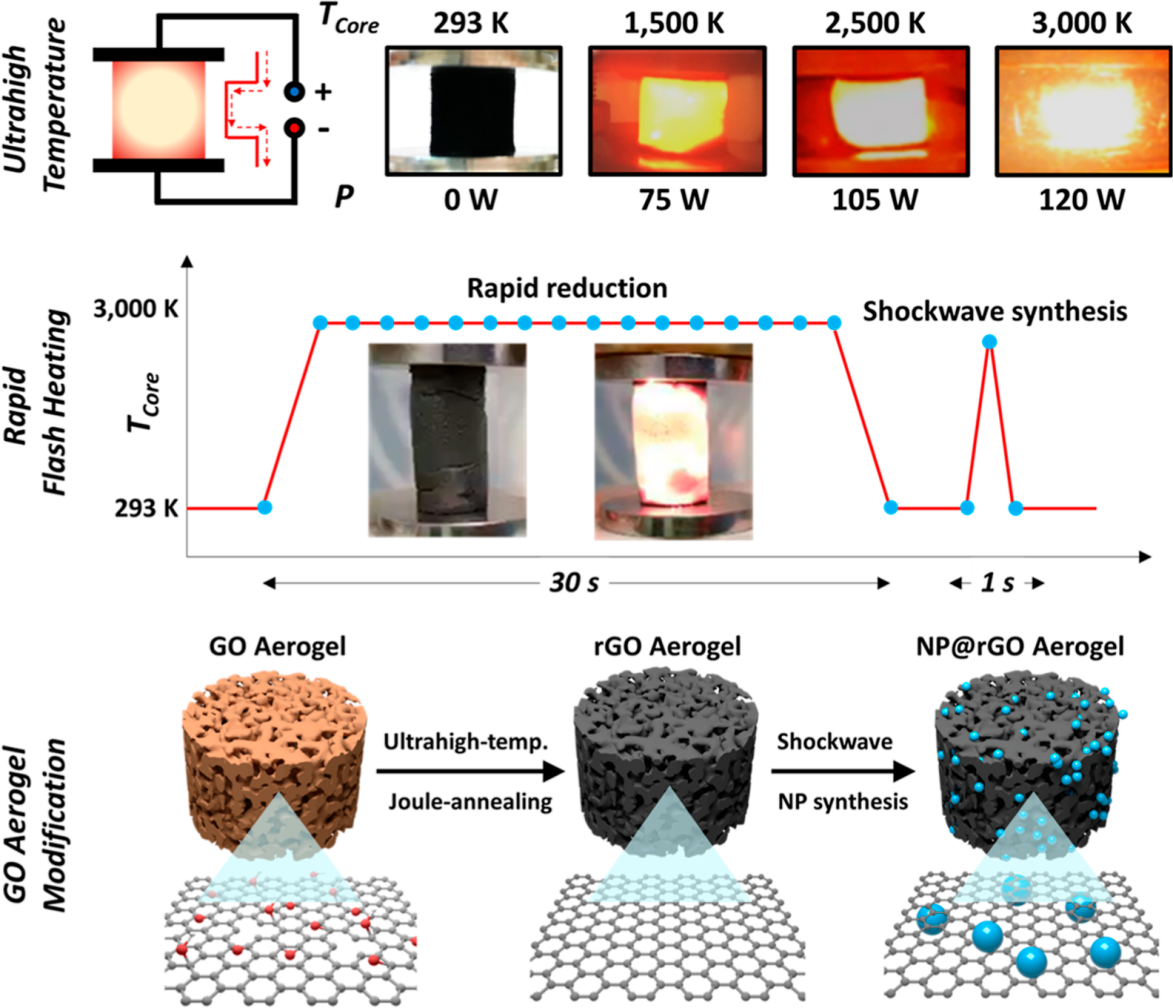
Overview Schematic of Ultrahigh-Temperature Flash Joule Heating of GO-Derived Aerogels and Its Application to GO Aerogel Modification in Terms of Rapid Joule Annealing and Shockwave Nanoparticle Synthesis

In contrast, Joule heating has the potential to enable a localized high-temperature aerogel heating approach at much lower energy costs and through a straightforward and flexible experimental design.^39^ High-temperature Joule heating of aerogels should also benefit from substantially improved control over the thermochemical conversion process due to the much shorter heating and cooling time scales.^1^ Despite these potential benefits, high-temperature Joule heating (>1500 K) has not yet been explored for 3D nanocarbon aerogels or foams, but has so far been limited to 1D and 2D macroscopic materials, such as nanocarbon-based fibers and films.^40,41^ In this study, high-temperature Joule heating is investigated for 3D nanocarbon aerogels for the first time, focusing on hydrothermal GO aerogels as a typical model system. Specifically, we exploit Joule heating as a multifunctional tool for the rapid thermochemical treatment of hydrothermal GO aerogels followed by pulsed ultrahigh-temperature, shockwave heating to synthesize embedded catalyst nanoparticles (Scheme 1). To this end, the fundamental characteristics of high-temperature aerogel Joule heating are investigated in depth to establish important structure–property relationships. Based on this understanding, high-temperature aerogel Joule heating will then be explored for a range of diverse purposes for the first time, including GO aerogel graphitization and size-controlled synthesis of aerogel-embedded functional metal and metal-oxide nanoparticles.

## Results and Discussion

### Electrothermally Driven Structural Evolution of the GO Aerogels

As an initial proof of concept, the structural evolution of a hydrothermally synthesized model GO aerogel under high-current Joule-heating conditions was investigated. Hydrothermal fabrication is one of the most widely utilized wet-chemical approaches for nanocarbon aerogel synthesis (Figure S1, Supporting Information).^42,43^ Hydrothermal aerogel fabrication typically imparts low levels of electrical conductivity [σ(GO) = 1.6 S·m^–1^, Table 1] due to chemical reduction and GO deoxygenation, making direct electrical heating methodologies feasible. However, restoration of graphitic crystallinity in hydrothermal GO aerogels is usually only partial, which leads to significant structural changes upon high-temperature Joule heating. To probe this microstructural evolution, a typical hydrothermal GO aerogel was Joule heated at a high power input of 120 W under a nitrogen atmosphere in a custom-made setup (Figure S2, Supporting Information) for 30 s (rGO_30s_) and 300 s (rGO_300s_), respectively.

**Table 1:** Structural and Chemical Characterization Data for an As-Synthesized GO Aerogel and GO Aerogels Joule Heated (Heating Current 10.1 A) for 30 s and 300 s, Respectively: (002) XRD Peak Position (2θ), XRD Peak Width (FWHM_(002)_), Corresponding *d*-Spacing (*d*_(002)_), Average Crystallite Domain Size (D_(002)_), and Average Number of Stacked Graphene Layers (*n*); Average Raman *I*_2D_/*I*_G_ Ratios (as Calculated from Raman Maps); Surface Oxygen Content (as Measured by XPS); Through-Volume Electrical Aerogel Conductivity (σ). rGO_Furnace_ Refers To a GO Aerogel Thermally Reduced via a Conventional Tube Furnace Treatment (1000 °C, 2 h, H_2_/N_2_ Atmosphere)

aerogel	2θ_(002)_ (deg)	fwhm_(002)_ (deg)	*d*_(002)_ (nm)	*D*_(002)_ (nm)	*n* (layers)	*I*_2D_/*I*_G_	oxygen (at %)	σ (S·m^–^^1^)
GO	24.80	8.287	0.359	1.0	4	0.22	22.8	1.6
rGO_30s_	25.90	3.167	0.344	2.7	9	0.3	0.7	81.5
rGO_300s_	26.20	1.505	0.340	5.7	18	0.77	0.6	93.2
rGO_Furnace_	26.11	2.752	0.341	3.1	10	0.32	2.0	13.5

Under these high current heating conditions, pronounced blackbody radiation is observed, with the temperature of the aerogel estimated to reach extremely high values of up to 3000 K (see also next section)—extremely challenging to produce via more conventional external heating methods. Despite the high current and temperature conditions, the aerogels remain structurally intact and exhibit no volume shrinkage. Achieving structural stability is challenging for many nanocarbon aerogels due to their extremely low solid volume fraction. This structural stability is also surprising in the context of previously observed destructive structural changes upon rapid heating of GO papers and films.^44−46^ The origins of the structural stability upon heating observed in our work likely originate in the synthetic conditions of GO aerogel fabrication. Specifically, chemical reduction of GO by ascorbic acid during aerogel synthesis has potentially removed more reactive functional group types from the GO structure, resulting in materials considerably less prone to the violent exothermic release of gaseous compounds upon fast heating. The extremely high porosity of our GO aerogels potentially also helps with mass transfer of suddenly released gaseous degradation products upon high-temperature heating, further mitigating structural disintegration during the Joule-heating process. Maintaining macroscopic structural integrity and avoiding monolith shrinkage highlights the efficacy of high-temperature Joule-heating treatments for (chemically reduced) GO aerogel materials, with Joule heating encouraging rGO intersheet cross-linking while maintaining extensive open porosity (Figures S3–S4, Table S1, Supporting Information).

**Figure 1:**
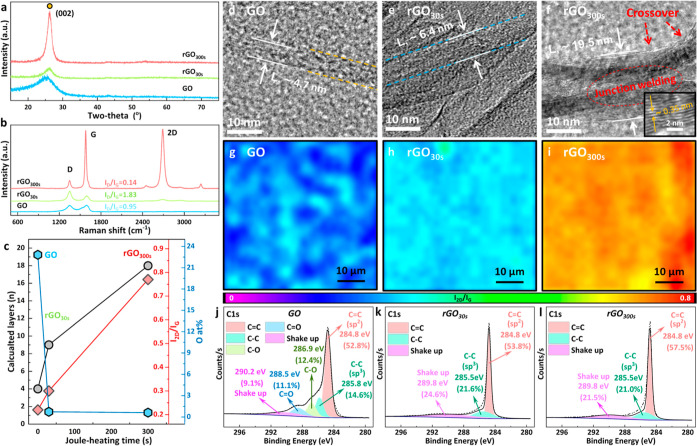
Evolution of GO aerogel graphitic structure and surface chemistry with Joule-heating duration: (a) XRD patterns, (b) Raman spectra, (d–f) TEM images, (g–i) Raman maps, and (j–l) C 1s XPS spectra of an as-synthesized GO aerogel and GO aerogels Joule heated at a heating current of 10.1 A for 30 s (rGO_30s_) and for 300 s (rGO_300s_), respectively. (c) Structural and chemical parameters as a function of Joule-heating duration: average number of stacked graphene layers (as determined by XRD); average *I*_2D_/*I*_G_ ratios (as determined from Raman maps) and surface oxygen content (as determined by XPS).

Upon Joule heating, aerogel envelope density decreases by half from 26 mg·cm^–3^ (GO) to around 13 mg·cm^–3^ (for both rGO_30s_ and rGO_300s_), indicating the removal of organic impurities (i.e., residues from the aerogel synthesis process) as well as GO deoxygenation. Despite the short heating durations, substantial improvements in graphitic crystallinity are observed, as indicated by a marked increase in electrical conductivity from 1.6 S·m^–1^ (GO) to 81.5 S·m^–1^ (rGO_30s_) and a substantial decrease in band gap from 0.09 eV (GO) to 0.01 eV (rGO_30s_, Figure S5, Supporting Information). Pronounced aerogel graphitization is confirmed by powder X-ray diffraction (XRD), Raman spectroscopy, and electron microscopy. XRD indicates a significant increase in the average number of stacked graphene layers from 4 layers (for the parent materials) to 18 layers (for the sample annealed for 300 s), as indicated by a significant width reduction of the graphitic XRD peak (Figure 1a). On a more localized scale, TEM imaging also shows a clear increase in rGO stack thickness for individual stacks upon Joule heating for 30 and 300 s (Figure 1d–f). The sharper contrast of the imaged graphitic lattice planes of the Joule-heated samples further suggests substantial improvement in graphitic crystallinity. TEM imaging also suggests that the high temperatures involved could have led to occasional sheet welding in the rGO_300s_ aerogels (Figure 1d–f, see also Figures S6–S10, Supporting Information); however, more detailed TEM studies are required for confirmation.

*I*_2D_/*I*_G_ Raman mapping of aerogel fragments, sampled from the aerogel center post-Joule heating (Figure 1g–i), confirms a marked reduction of rGO defectiveness on the mesoscale, as indicated by the transition from an average *I*_2D_/*I*_G_ ratio of ∼0.22 (GO) to a relatively high average *I*_2D_/*I*_G_ of ∼0.77 (rGO_300s_).^47^ This is further confirmed by the sharp and symmetric G and 2D peaks in the Raman spectra (Figure 1b). It is worth noting that the 30 and 300 s Joule-annealing treatment not only reduces the average defect level but also results in the formation of structurally more homogeneous samples, as indicated by a more uniform color distribution in the Raman maps (Figure 1h–i). In terms of chemical changes, X-ray photoelectron spectroscopy (XPS) shows a substantial loss of oxygen surface groups from around 20 at % oxygen to <1 at % O for both 30 and 300 s Joule-annealing treatments (Figure 1j–l, Table 1), also evidencing that carbon oxidation can be successfully avoided despite the high temperatures reached.

It is worth noting that GO deoxygenation can be achieved at even shorter time scales, with XPS indicating a dramatic reduction in oxygen concentrations to 1.5 at. % after only 10 s high-temperature Joule heating (Figure S22, Table S3, Supporting Information). Short, 10 s Joule heating, also induces some improvements in graphitic crystallinity, as indicated by Raman spectroscopy (Figure S23, Table S4, Supporting Information), with further ongoing improvements in graphitization at the 30 and 300 s time scales. These observations indicate that high-temperature Joule heating induces rapid GO deoxygenation, followed by slower and gradually stabilizing annealing processes at longer heating durations, in line with in situ TEM studies of GO reduction under Joule-heating conditions.^48^

Even though the employed Joule-heating durations are relatively short, the level of aerogel graphitization achieved is comparable to a much longer, conventional graphitization treatment carried out in a tube furnace (1000 °C, 2 h).^49^ The furnace-graphitized aerogel sample shows similar structural and chemical material characteristics as the aerogel sample treated via high-current Joule annealing for 30 s (Table 1). However, the energy consumption of a 30 s Joule-annealing treatment (∼1 × 10^–6^ kW·h) is orders of magnitude lower than that of a conventional furnace treatment (∼5 kW·h) due to the short time scales and comparatively low applied voltages employed.

Joule annealing for 300 s achieves even higher graphitic crystallinity than the conventional furnace treatment (Table 1), while energy consumption still remains comparatively low (∼1 × 10^–5^ kW·h). These findings highlight that aerogel Joule heating to ultrahigh temperatures (up to *T*_core_ ∼ 3000 K) provides a straightforward tool for aerogel graphitization and annealing. Advantages of this Joule-annealing approach include fast process speeds and much improved energy efficiency as well as a comparatively straightforward and flexible experimental setup. In addition, the degree of graphitization can be easily controlled through the Joule-heating duration at relatively short time scales (Figure 1b,c).

### Ultrahigh-Temperature Electrothermal Heating Characteristics of the rGO Aerogels

To gain a better understanding of the ultrahigh-temperature aerogel heating characteristics, Joule heating of a typical rGO aerogel was studied across an extremely wide range of high electrical power input conditions. Key heating parameters such as equilibrium aerogel surface and core temperatures as a function of power input as well as heating and cooling rates were determined. As a model system, the rGO_30s_ aerogel from the previous section was selected as it shows structural properties, comparable to rGO aerogels produced through conventional furnace heating methods (Table 1). For the remainder of this paper, the rGO_30s_ aerogel will therefore be simply referred to as the rGO aerogel.

**Figure 2:**
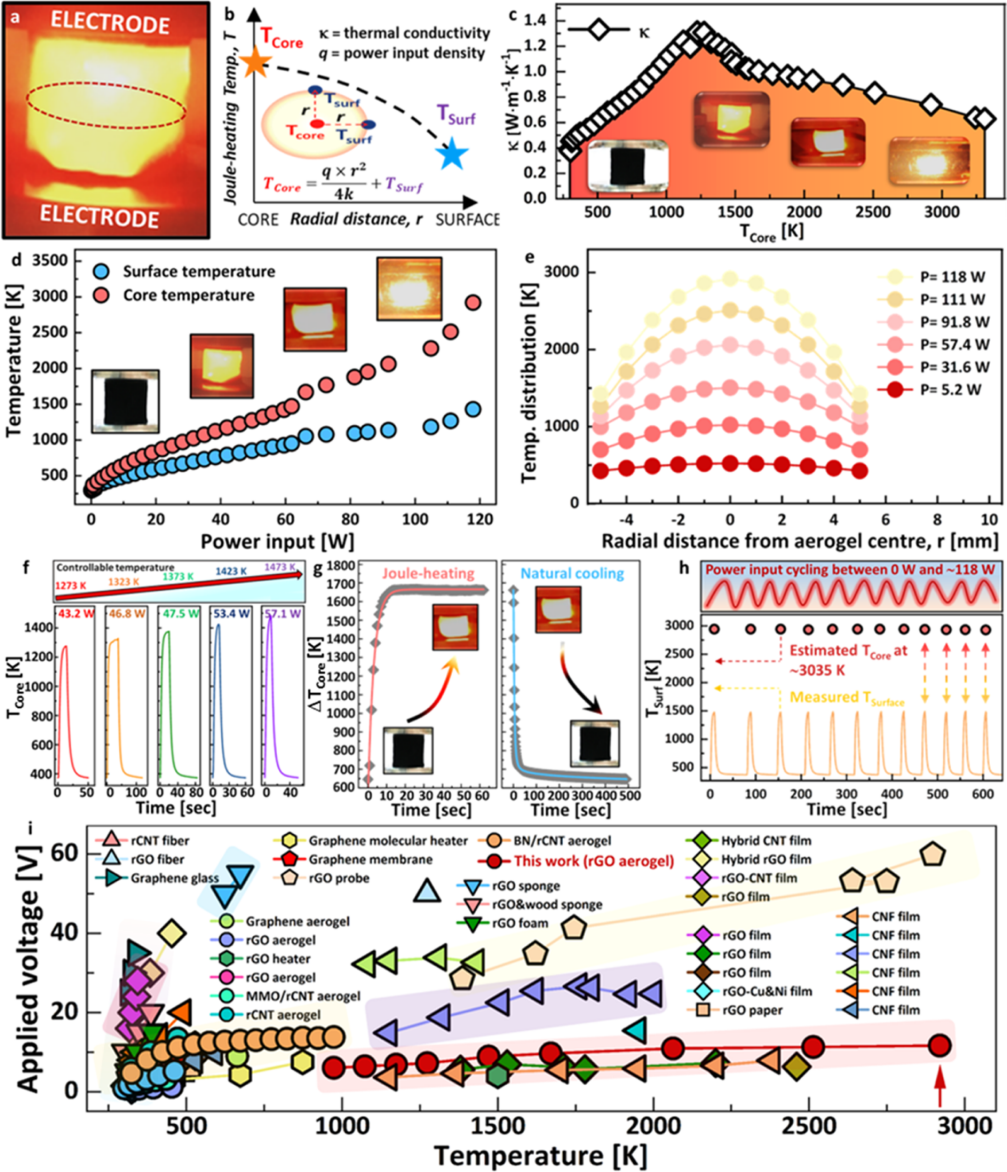
(a) Digital photograph of a Joule-heated GO aerogel at a power input of 120 W, emitting blackbody radiation. (b) Schematic core-to-surface temperature gradient for the Joule heating of a cylindrical aerogel monolith, based on 1D heat transfer. (c) Thermal rGO aerogel conductivity as a function of rGO aerogel core temperature. Insets: rGO aerogels at different *T*_core_, emitting blackbody radiation at different intensities and wavelengths. (d) rGO aerogel core temperature and surface temperature as a function of electrical power input. (e) Estimated Joule-heating temperature gradients across the cross-sectional aerogel diameter, at different electrical power inputs. (f) Control of high Joule-heating temperatures through control of power input. (g) Estimated core temperature change as a function of time for ultrahigh Joule heating (power input 100 W, *T*_surf_ ∼ 1317 K; *T*_core_ ∼ 1932 K) and for subsequent natural cooling after heating current switch off. (h) Repeatable ultrahigh thermal cycling of rGO aerogel at 120 W electrical power input (aerogel surface temperatures measured optically; aerogel core temperatures estimated). (i) Comparison of ultrahigh rGO Joule heating with other nanocarbon-based Joule heaters reported in the literature.

At the highest high-current conditions (10 A, 11.8 V, and 118 W), the rGO aerogel showed a bright and striking orange-white glow (Figure 2a), indicating heating to ultrahigh temperatures, resulting in emission of blackbody radiation in the visible spectrum.^50^ The equilibrium surface temperature of the orange-glowing aerogel was measured to be around 1430 K, an extremely high temperature value that is very challenging to reach via more conventional external heating methodologies. Joule-heating temperatures in the interior of the 3D aerogel monoliths are likely to be even higher than the measured surface temperature.

However, temperature measurements at the aerogel core are extremely challenging as neither conventional thermocouples nor optical methods can be used in the ultrahot aerogel interior. Therefore, core temperatures were estimated via an indirect methodology, specifically via extrapolation of the aerogel core temperatures from measurements of the more accessible aerogel surface temperatures (Figure 2b). Briefly, the aerogel surface and core temperature are linked via the aerogel’s thermal conductivity, κ (Figure 2b), assuming 1D heat conduction.^1^ Knowledge of κ therefore allows us to estimate the aerogel’s radial thermal gradient, i.e., the aerogel’s Joule-heating temperature, T, as a function of distance, r, from the aerogels core at a given power input, *q*. As κ is itself temperature-dependent, it needs to be estimated for different temperatures.^51^ As is characteristic for graphitic carbon materials, the thermal dependence of the aerogels’ κ values displays a typical two-branched Umklapp scattering profile across the extremely wide temperature window probed in this study (Figure 2c). Using surface temperature measurements and established fitting methodologies (Figures 2b, S11–S14, Supporting Information),^51^ the dependence of the aerogel’s thermal conductivity was quantitatively estimated (Figure 2c). The resulting κ values were then used to estimate the core temperature from the surface temperature and establish the evolution of equilibrium core temperature as a function of Joule-heating power input (Figure 2d).

Based on this approach, it is estimated that, at a power input of 120 W, an ultrahigh temperature of around 3000 K is reached in the center of the aerogel monolith, an extremely high value close to the melting point of graphitic carbon allotropes.^52^ This steady-state core temperature is almost an order of magnitude higher than for many previously reported aerogel heating studies on GO-derived aerogels (Figure 2i, Table S2, Supporting Information),^1,2,4,29,51,53−69^ mainly due to the high power inputs employed here. It is also worth noting that ultrahigh aerogel heating was achieved here at comparatively low voltage inputs (10 V or lower, Figure 2i), due to the relatively high graphitic quality and corresponding high electrical conductivity of rGO aerogels (here, σ_rGOaeroel_ = 81 S m^–1^). More generally, the findings show that 3D structured rGO aerogels can be Joule heated to similarly high temperatures as previously demonstrated for 2D nanocarbon films and filaments (Figure 2i).^2,51^ Specific ultrahigh aerogel temperatures can be readily and repeatedly selected through simple control of the electrical input power (Figure 2f). A distinct difference from nanocarbon filament Joule heating is, however, that the three-dimensionality of the rGO aerogel monoliths can give rise to marked surface-to-core thermal gradients. While differences between aerogel surface and core temperature are relatively small for low and medium heating regimes, they can be substantial at ultrahigh temperatures (Figure 2e). For practical applications, insulation of the outer aerogel surface provides a straightforward mitigation approach against these temperature gradients.^1^

Crucially, Joule heating also enables extremely fast heating of the aerogels (flash heating).^10^ For example, aerogel heating from ambient to ultrahigh temperature (*T*_core_ = 1940 K; Δ*T*_core_ = 1670 K) occurs in less than 10 s (Figure 2g). Initial Joule-heating rates reached at least 300 K·s^–1^. Estimation of the heating rates is limited by the lag of the thermocouples and might be even higher as even within 1 s high-current Joule-heating blackbody radiation (i.e., temperatures >500 °C) is observed. Cooling kinetics (i.e., natural cooling through heat loss to the environment after switching off the electrical heating current) are also fast, especially cooling from ultrahot to moderate temperatures, which also occurs at rates of around 200 K·s^–1^. Cooling kinetics from moderate to ambient temperatures is somewhat slower, potentially due to the slow convection of heated gas out of the aerogel interior (Figure 2g). Nevertheless, cooling rates remain remarkably high compared to more conventional furnace approaches. These fast heating and cooling kinetics allow for very stable thermal cycling, as demonstrated for Joule-heating cycling at 1473 K surface temperature, corresponding to an estimated core temperature of around 3035 K (Figure 2h).

### Flash Joule Heating for Thermo-chemical Nanoparticle Synthesis

These remarkable aerogel heating characteristics, achievable through high-current Joule heating, can be exploited for chemical transformations beyond the GO graphitization treatments, investigated in the first section. Flash Joule heating to ultrahigh temperature was explored for the controlled thermochemical transformation of molecular precursors to functional nanoparticles, in effect utilizing the porous rGO aerogels as fast and energy-efficient nanoscale furnaces. Conventionally, thermal precursor conversion treatments are carried out at moderate to high temperatures (450–1150 K) via external heating treatments.^34,70,71^ However, external heating can be energy-intensive and difficult to control due to the relatively long heating time scales (>1 h) as well as relatively slow cooling rates.^40^

**Figure 3:**
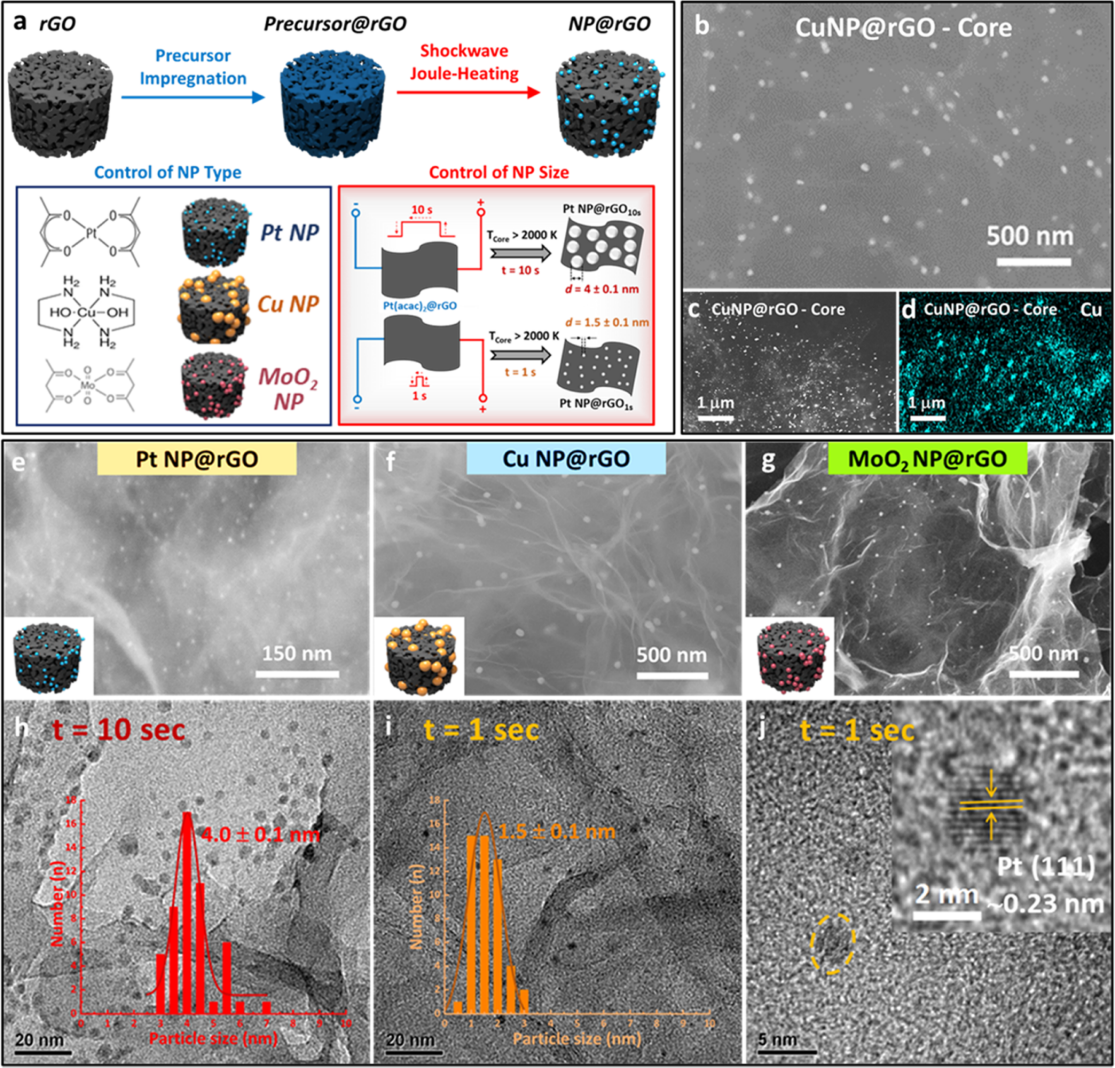
(a) Fabrication of rGO-aerogel-supported nanoparticles via aerogel wet-impregnation and subsequent aerogel shockwave Joule heating. Schematic, depicting control of nanoparticle type and size via loading of different coordination compound precursors and control of flash Joule-heating duration, respectively. (b) SEM image of Cu NP@rGO aerogel sampled from the aerogel center. SEM image (c) and corresponding EDX mapping (d) of the Cu NP@rGO aerogel sampled from the aerogel center. Higher-resolution SEM images of (e) Pt NP@rGO aerogel, (f) Cu NP@rGO aerogel, and (g) MoO_2_ NP@rGO aerogel, synthesized via the flash Joule-heating method. TEM images and particle size distributions of Pt NP@rGO aerogel produced via (h) 10 s flash Joule heating and (i) 1 s flash Joule heating, respectively. (j) High-resolution TEM image of rGO-supported Pt NP produced via 1 s flash Joule heating.

To demonstrate the applicability of flash Joule heating for aerogel functionalization, the synthesis of Pt nanoparticles (important chemical and electrochemical catalysts) embedded within the aerogel framework was studied. To this end, preconditioned rGO aerogels were wet-chemically impregnated with a Pt nanoparticle precursor solution [Pt(acac)_2_ in chloroform], followed by aerogel drying to homogeneously embed the precursor (Figure 3a)—a well-explored and commonly utilized approach in the literature.^35^ The precursor-decorated aerogels (nominal Pt loading 5 wt %) were then shockwave Joule-heated in an inert atmosphere, i.e., Joule-heated to ultrahigh temperatures (*T*_core_ ∼ 2200 K) for very brief durations (<10 s) under high current conditions (100 W), allowing for very fast precursor decomposition and conversion into nanoparticles (Figure 3a). SEM and TEM imaging show extremely fine Pt nanoparticles with a narrow size distribution (*d*_NP_ = 4.0 ± 0.1 nm, Figure 3h), uniformly distributed across the aerogel network (Figure 3e). Successful aerogel functionalization with Pt nanoparticles is further confirmed by XPS (Figure S17 and Supporting Information). To demonstrate the scope of Joule-heating-based aerogel functionalization, aerogels were infused with alternative molecular nanoparticle precursors [Cu(en)_2_(OH)_2_; MoO_2_(acac)_2_, Figure 3a]. Precursor compounds were selected on the basis of both their molecular structure (coordination compounds containing flat subunits that promote interaction with and adsorption by the graphitic rGO surfaces) and their solubility (compounds exhibiting appreciable solubility in solvents that are capable of rGO aerogel wetting and impregnation). Following precursor impregnation, aerogels were shockwave Joule-heated at a power input of 100 W for 1 s (*T*_core_ ∼ 2200 K) to induce thermochemical nanoparticle formation. XRD, energy-dispersive X-ray spectroscopy (EDX), and XPS evidence successful aerogel functionalization with metallic Cu nanoparticles and MoO_2_ nanoparticles, respectively. Electron microscopy (Figures 3f–g, S18–S21, Supporting Information) shows nanoparticles of small size and narrow size distribution (*d*_Cu_ = 41.5 ± 0.9 nm; *d*_MoO_2__ = 16.4 ± 1.6 nm) that are uniformly distributed throughout the whole aerogel monolith. These results demonstrate the broad applicability of the shockwave Joule-heating approach for effective and well-controlled modification of aerogels with a wide range of functional nanoparticles.

To further confirm 3D uniformity, Cu nanoparticle-decorated rGO aerogels were imaged by SEM at different locations within the monolith. Aerogel fragments sampled from the aerogel core and aerogel surface, respectively, show nanoparticles of very similar size and size distribution, with a smaller than 10% difference in average particle size at the different locations (Figures 3b–d, S18, Supporting Information). Importantly, the similar nanoparticle sizes at the aerogel core and surface highlight that the temperature gradients mentioned in the previous section do not negatively impact the nanoparticle formation process. This observation suggests that the very short heating durations (as well as the fast cooling rates) limit thermally activated migration of molecular metal intermediates across the graphitic surface. This finding also highlights that the combination of ultrahigh temperatures (ensuring extremely rapid precursor decomposition and particle formation) and short heating durations (<10 s, limiting particle sintering) is key to ensure functionalization homogeneity of the 3D aerogel systems.

Crucially, Joule-heating duration can also be exploited to reduce nanoparticle size, an important factor, especially for catalytic applications where control over nanoparticle size is one of the key structural parameters to control functional activity. Reducing the Joule-heating duration from 10 to 1 s substantially reduces the average Pt nanoparticle size to an extremely fine 1.5 nm, as observed by TEM imaging (Figures 3i,j, S15–S16, Supporting Information). Despite this short heating duration, the formed nanoparticles are highly crystalline, as indicated by the characteristic Pt(111) lattice spacings observed by HR-TEM (Figure 3j). The substantially reduced particle size is likely due to minimized precursor aggregation over the very short heating time while still providing sufficient energy to initiate precursor transformation. Further reduction of heating time into the microsecond regime in combination with pulsed heating approaches might allow for further size reduction or even formation of single atomic sites.

## Conclusions

High-temperature Joule heating was investigated, for the first time, for the thermo-chemical modification of 3D, highly porous nanocarbon aerogels. Hydrothermally synthesized, chemically reduced GO aerogels can be readily heated to ultrahigh temperatures (up to *T*_core_ ∼ 3000 K) at comparatively low input voltages (<10 V), using a straightforward and flexible setup. High-temperature aerogel Joule heating is shown to be very fast, with heating rates readily exceeding 300 K·s^–1^. In contrast to the Joule heating of 2D nanocarbon films, core-to-surface temperature gradients are observed within the 3D aerogel monoliths, especially in the ultrahigh heating temperature regime. These temperature gradients are shown to have no significant impact on applications, such as Joule-heating-based nanoparticle formation, but should be considered when developing new high-temperature Joule-heating methodologies for functional aerogels, such as thermal polymer depolymerization or thermal aerogel regeneration approaches. The practical utility of high-temperature flash Joule heating of rGO aerogels is demonstrated for high-quality graphitic annealing and for thermochemical, in situ synthesis of aerogel-embedded functional nanoparticles (Pt, Cu, and MoO_2_). Due to the fast heating rates, these high-temperature treatments can be carried out at very short time scales (<10 s), rendering this approach orders of magnitude more energy efficient compared to more conventional external high-temperature heating methods. Importantly, the short heating durations in combination with the excellent cooling efficiency of rGO aerogels allow them to not only rapidly initiate but also rapidly arrest high-temperature processes, crucial to avoiding unwanted thermal effects, such as particle sintering or temperature-gradient-induced structural inhomogeneities.

These findings lay a solid foundation to develop advanced flash Joule-heating methodologies for high-temperature aerogel modifications, e.g., for the synthesis of aerogel-embedded high-entropy-alloy nanoparticles, the functionalization of nanocarbons with highly functional single atomic sites, or the fabrication of 3D thermoelectric materials. Joule heating also offers unique opportunities for in situ thermochemical aerogel treatment, i.e., for aerogel treatments directly at the location of their application. In situ aerogel modification through direct Joule heating (e.g., in situ graphitization, functional reactivation, or nanocatalyst functionalization) would be especially valuable in the context of emerging new continuous process technologies such as aerogel-based (electro)catalytic flow-based chemical synthesis or purification methodologies.

## Methods

### Materials

GO (spray-dried GO flakes) was purchased from William Blythe Limited (UK). l-Ascorbic acid, bis(ethylenediamine)copper(II) hydroxide [Cu(en)_2_OH_2_], platinum(II) acetylacetonate [Pt(acac)_2_], and bis(acetylacetonato)dioxomolybdenum(VI) [MoO_2_(acac)_2_] were obtained from Sigma-Aldrich (UK). HPLC water was provided by Fisher Scientific (UK). Chloroform was supplied by Fisher Scientific, UK. All chemicals were used as received.

### Synthesis of Hydrothermal GO Aerogels

GO flakes (0.16 g) and l-ascorbic acid (0.64 g) were added into HPLC water (40 mL) at a weight ratio of 4:1, followed by ultrasonication (10 min per cycle, 9 cycles in total) to form a stable GO dispersion. 7.5 mL of the resulting dispersion was transferred to a sample vial, sealed, and hydrothermally treated at 50 °C for 24 h to form a GO hydrogel. The resultant hydrogel was solvent exchanged in HPLC water to remove the majority of the l-ascorbic acid reducing agent. Four solvent exchanges were carried out, each soaking the GO hydrogel monolith in HPLC water for 1 h (with the resulting hydrogel retaining around 10 wt % ascorbic acid relative to the GO weight). The hydrogel was then frozen in liquid nitrogen and freeze-dried to obtain the GO aerogel (Figure S1, Supporting Information). The resulting aerogel monoliths had a cylindrical shape (diameter ∼10 mm; height ∼10 mm).

For the rGO_Furnace_ aerogel (mentioned in Table 1), the hydrothermal GO aerogel was thermally treated in a tube furnace (Carbolite Gero Ltd., UK) at 1000 °C for 2 h under a reducing H_2_/N_2_ atmosphere (5% H_2_).

### High-Temperature Joule Heating

All aerogel Joule-heating measurements were carried out under nitrogen within an airtight Perspex container with gas inlets. Samples were electrically contacted within the container through a custom-made sample holder consisting of two steel plate electrodes attached to two movable, heat-resistant alumina holder blocks (Figure S2, Supporting Information). Samples were contacted directly with the steel electrodes without the need for any conductive additive. Electrical current and voltage were controlled using a portable power source (ES-PS, 3032-10 B). Prior to the Joule-heating experiments, the container was flushed with pure nitrogen gas for 30 min to eliminate any remaining air. The aerogel core and surface temperatures were determined by two thermocouples (0.25 mm of diameter, K-type grounded tip, TJC 120 Series, Omega, UK), with one thermocouple inserted into the aerogel center and one thermocouple contacting the aerogel surface (Figure S2, Supporting Information). To measure ultrahigh temperatures, beyond the temperature limitations of the thermocouples (>1200 °C), an IR thermometer was used to determine the aerogel surface temperatures from the emitted blackbody radiation. Stepwise Joule-heating experiments were carried out by increasing input current in increments followed by equilibration of each step for 20 min, unless otherwise stated. Process parameters, such as temperature, current, and voltage, were continuously recorded through data loggers (EL-USB-TC, Lascar Electronics).

### Electrothermal Shockwave Synthesis of NP@rGO Aerogels

For aerogel functionalization with metallic Pt nanoparticles, a rGO_30s_ aerogel monolith was soaked in 10 mL of platinum(II) bis(acetylacetonate) solution [0.05 mM Pt(acac)_2_ in chloroform] for 24 h under constant orbital shaker agitation. The aerogel was then recovered from the solution and left to dry under ambient conditions for 24 h. The dried aerogel was subsequently Joule heated at an electrical current of *I* = 10.1 A for either 1 or 10 s to yield the Pt NP@rGO_1s_ aerogel and Pt NP@rGO_10s_ aerogel, respectively. Similarly, the MoO_2_ NP@rGO aerogel and Cu NP@rGO aerogel were fabricated following the same experimental procedure as above, using a bis(acetylacetonato)dioxomolybdenum(VI) solution [1 mM MoO_2_(acac)_2_ in chloroform] and a bis(ethylenediamine)copper(II) hydroxide solution [1 mM Cu(en)_2_(OH)_2_ in water], respectively.

### Material Characterization

XRD patterns were measured on a Bruker D2 Phaser diffractometer using Cu Kα radiation, with a scanning angle range from 5° to 90°. Specifically, a cylindrical aerogel monolith was horizontally cut into circular, 5 mm thick pieces. The aerogel piece, closest to the aerogel monolith middle, was compressed into a flat, disc-like specimen and then clamped onto a flat silicon XRD sample holder. Raman spectroscopic analysis was conducted on a Renishaw InVia confocal Raman microscope with an excitation laser wavelength of 532 nm between 400 and 4000 cm^–1^. Raman mapping was performed on the same equipment at an excitation laser wavelength of 697 nm, between 1300 and 3000 cm^–1^, scanning continuously on a focused area of 50 μm × 50 μm. For the Raman measurements, an aerogel was cut in half, and a flake (∼4 mm^2^) was sampled from the aerogel center. The flake was compressed between two glass slides to form a flat paper-like specimen which was then placed onto the Raman sample holder for measurements. Scanning electron microscopy (SEM) was carried out using a Nova NanoSEM 450 scanning electron microscope at an accelerating voltage of 3 keV. For SEM sample preparation, an aerogel flake was again sampled from the aerogel center and fixed onto aluminum stubs via conducting carbon tape. Considerable care was taken to avoid flake compression to minimize changes in the aerogel microstructure. EDX was carried out on the same SEM instrument at an accelerating voltage of 18 keV. Transmission electron microscopy (TEM) images were taken on an FEI Tecnai F30 scanning transmission electron microscope at an accelerating voltage of 300 keV. Several small flake specimens (∼1 mm^2^) were sampled from the aerogel core and aerogel surface (midpoint of the aerogel cylinder sidewall), respectively. Two or three flakes were bath-sonicated in ethanol (5 mL), drop-cast onto a copper grid, and dried at room temperature. XPS was carried out on a Thermo Scientific K-Alpha X-ray photoelectron spectrometer; high-resolution scans were collected at a pass energy of 30 eV and a step size of 0.1 eV. The binding energies were referenced to the C 1s peak of adventitious carbon at 284.8 eV. For the XPS measurements, larger flakes (∼4 mm^2^) were sampled from the aerogel center, dispersed in ethanol (5 mL), then drop-cast onto a flat silicon substrate, followed by drying within a desiccator at room temperature. Prior to characterization, all aerogel samples were stored in a desiccator, with samples being directly transferred after freeze-drying (last step of aerogel synthesis) or heating treatments.

## Supporting Information Available

Schematics of GO aerogel synthesis procedures, digital photos of the ultrahigh-temperature Joule-heating setup, Joule-heating parameters, contact angle, BET analysis, SEM and TEM images, EDX mapping, Arrhenius model fitting analysis, estimation of Joule-heating temperatures for monolithic aerogels, thermogravimetric analysis, Raman map, XPS spectrum analysis, XRD patterns, Raman spectrum analysis, and summary of nanocarbon-based Joule heaters (PDF)
