# Supplementary material for: Electrothermal Transformations within Graphene-Based Aerogels through High-Temperature Flash Joule Heating
Source: J Am Chem Soc. 2023 Dec 30;146(1):159–69. doi: 10.1021/jacs.3c06349 (PMC10786031; doi:10.1021/jacs.3c06349)
Supplement: Supplementary file 1 — ja3c06349_si_001.pdf [file ja3c06349_si_001.pdf]

Supporting Information

Electrothermal Transformations within Graphene-Based Aerogels through High-Temperature Flash Joule Heating

Dong Xia[†], Jamie Mannering[†], Peng Huang[‖], Yifei Xu[Φ], Qun Li[Π], Heng Li[Ψ], Yi Qin[‡], Alexander N. Kulak[†] and Robert Menzel[†*]

[†] School of Chemistry, University of Leeds, Leeds, LS2 9JT, UK

[‖] Department of Materials, University of Manchester, Manchester, M13 9PL, UK

[Φ] State Key Laboratory of Molecular Engineering of Polymers, Department of Molecular Science, Fudan University, Shanghai, 200438 China

[Π] School of Chemistry and Chemical Engineering, Chongqing University, Chongqing, 400044, China

[Ψ] Key Laboratory of Estuarine Ecological Security and Environmental Health, Tan Kah Kee College, Xiamen University, Zhangzhou, 363105, China

[‡] Department of Engineering Science, University of Oxford, Oxford, OX1 3PJ, United Kingdom

Materials characterization

Thermogravimetric analysis-Fourier transform infrared spectroscopic analysis (TGA-FTIR) was performed on a hyphenated thermogravimetric analyser (Q20, TA instruments) and Fourier Transform Infrared Spectrometer (Nicolet iS10, Thermo Fisher Scientific), with a ramping rate of 10 ^{o}C/min from 20 to 800 ^{o}C, in air atmosphere. The aerogel samples (~20 mg) were ground into flakes in the mortar prior to the thermal decomposition, in order to prevent the ultralight aerogel from drifting away.

Contact angles of the aerogels were tested using a drop-shape analysis device (OCA 25, Dataphysics UK). The top surface of aerogels was polished to horizonal for the contact angle analysis.

Brunauer-Emmett-Teller (BET) surface area measurements were conducted on a Micromeritics TriStar 3000 instrument. The samples (~100 mg) were degassed in nitrogen atmosphere at 110 ^{o}C for 3 h before analysis. The nitrogen adsorption/desorption isotherms were measured at 77 K. The pore diameter distribution of the samples was determined from the desorption isotherm using the Barrett-Joyner-Halenda (BJH) method.

Flash Joule-heating of GO-derived aerogels

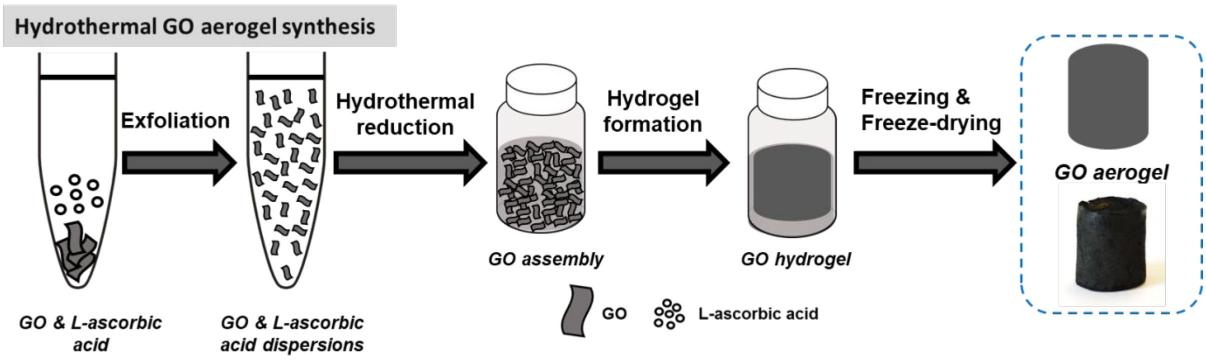

Figure S1. Schematic depiction of hydrothermal synthesis process to produce as-synthesised GO aerogels.

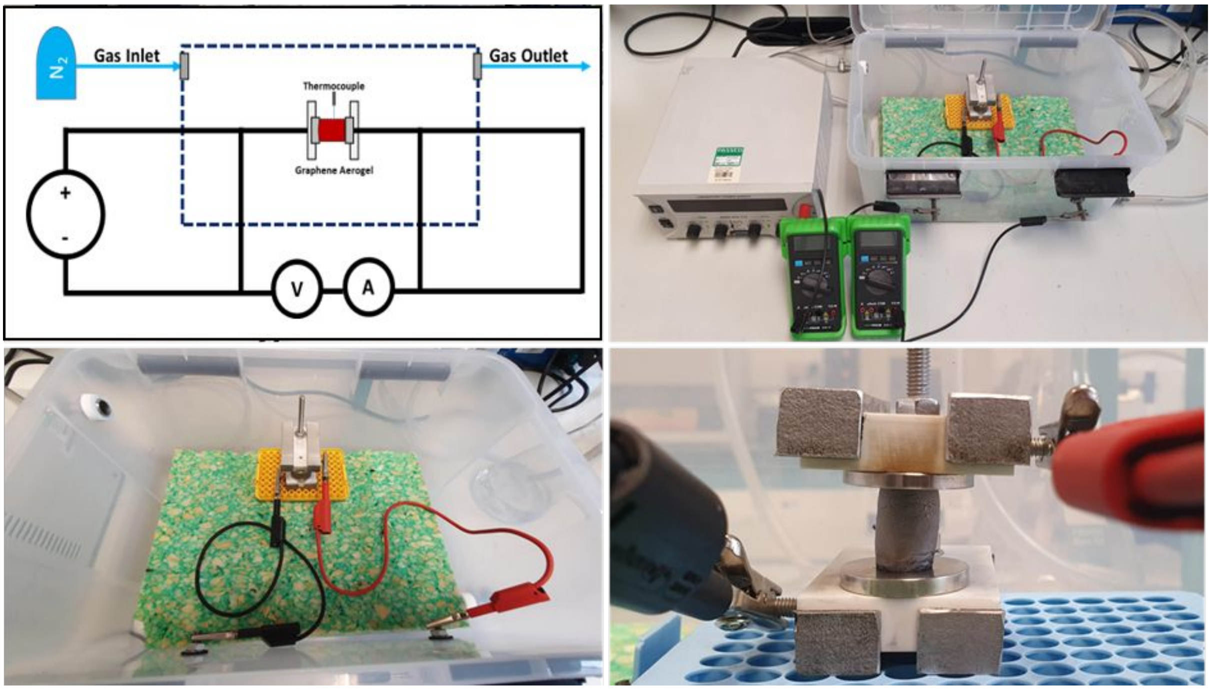

Figure S2. Schematic and photographs of high-temperature Joule-heating set-up

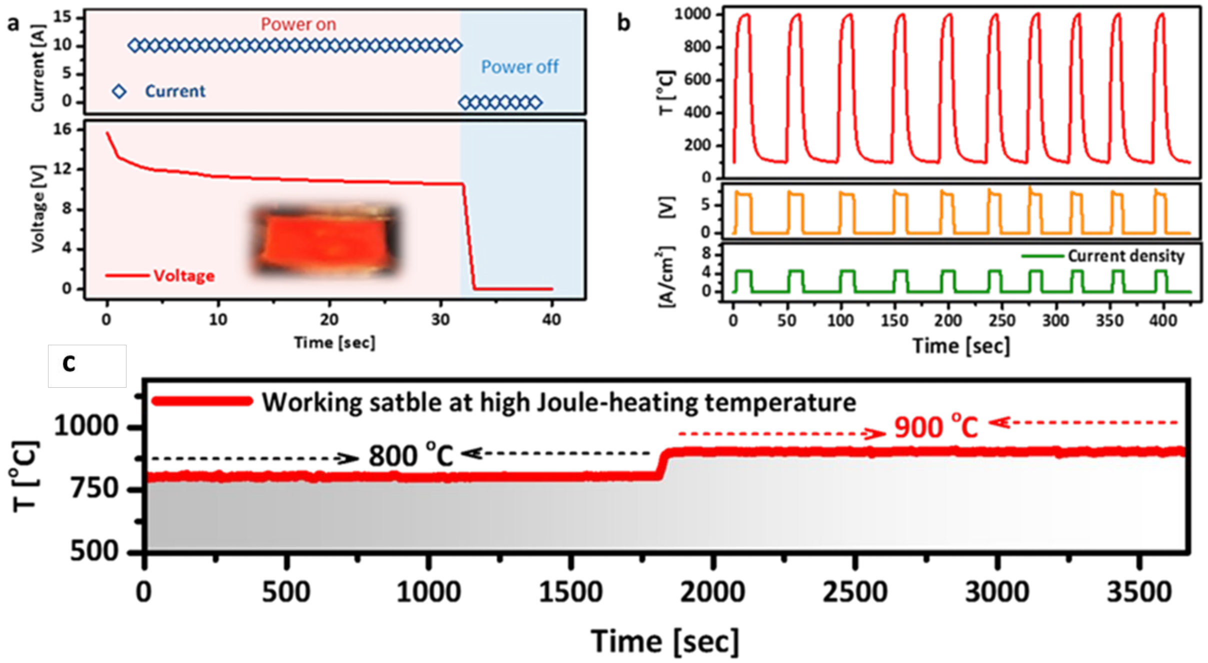

Figure S3. rGO$_{30s}$ aerogels obtained via Joule-heating. (a) Current and voltage profile for the ultrahigh temperature Joule-heating of a GO aerogel for 30 s. Insert is a digital image of an aerogel monolith during heating, emitting black-body radiation. (b) Surface temperature, Joule-heating voltage and current density for the thermal cycling of a rGO$_{30s}$ aerogel over 10 thermal cycles. (c) Highly stable heating if rGO$_{30s}$ aerogels to 800°C and 900°C, respectively.

The as-synthesized GO aerogel was carried out via Joule-heating, using the setup depicted above (Figure S2). For ultrahigh-temperature Joule-heating, aerogels were Joule-heated at a constant heating current of 10.1 A under nitrogen atmosphere for 30 s (Figure 3a). During this treatment, the emission of intense red-coloured black body radiation. Thermocouple measurements at the aerogel surface confirm that temperatures of at least 1200 °C is reached. Over the pre-conditioning duration, a clear decrease in heating voltage is observed within the first 5 s. This sudden drop in aerogel resistance is attributed to GO deoxygenation and impurity removal (i.e. thermal degradation of ascorbic acid residues remaining from the hydrothermal aerogel synthesis) at the high temperatures reached. Over the remaining 25 s of the preconditioning, a further, small but continuous decrease in heating voltage is observed, indicating a slight improvement in graphiticity over this duration.

The resulting, rGO$_{30s}$ aerogels are extremely stable under the high-temperature conditions explored in this work. To demonstrate this point, a rGO$_{30s}$ aerogel was repeatedly Joule-heated to high temperatures (Figure S3b). Specifically, the same high aerogel temperature (T_{surf}= 1000 °C) was accurately reached over 10 Joule-heating cycles, using the same current and voltage input in each cycle. This reliable high-temperature cycling behaviour confirms aerogel stability during high-temperature flash Joule-heating treatments, such as the thermo-chemical

nanoparticle synthesis described in the main text. At the same time, the preconditioned aerogels can be heated very stably to 800°C and 900°C in inert atmosphere for long durations (>30 min, Figure S3c). This high-temperature stability is important for aerogel applications beyond flash heating, e.g. in high-temperature chemistry, temperature-swing regeneration of aerogel catalysts and sorbents and aerogel-based high-temperature sensors.

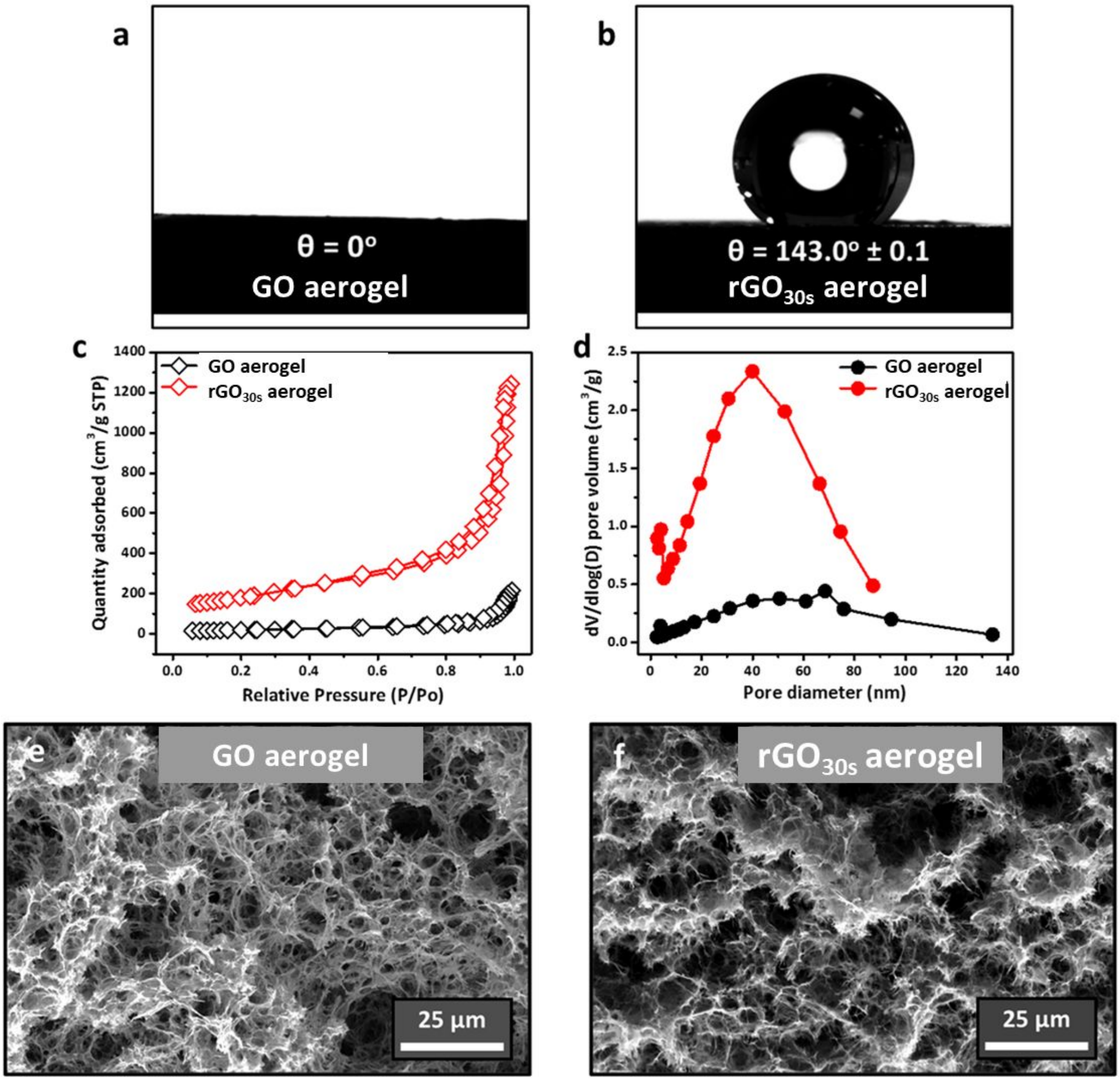

Figure S4. Materials characterisation of pre-conditioned aerogels. (a-b) Water contact angles of GO aerogel and rGO_{30s} aerogel. (c-d) Nitrogen adsorption-desorption isotherms and pore size distributions of the GO aerogel and rGO_{30s} aerogel. (e-f) SEM images of internal microstructure for the GO aerogel and rGO_{30s} aerogels.

Table S1. Physico-chemcial characterisation of GO aerogel and rGO_{30s} aerogel, including electrical conductivity (σ), specific surface area (SSA), meso-pore volume ($V_{Meso\text{-}porosity}$), and micro-pore volume (V_{micro}).

Name	σ ($S\cdot m^{-1}$)	SSA ($m^2\cdot g^{-1}$)	$V_{Meso\text{-}porosity}$ ($cm^3\cdot g^{-1}$)	V_{micro} ($cm^3\cdot g^{-1}$)
**GO aerogel**	1.6	68.1	0.34	0.0018
**rGO_{30s} aerogel**	81.5	634.3	1.93	0.0224

In terms of textural properties, N_2 adsorption/desorption measurements (Figure S4c, Table S1) indicate that aerogel pre-conditioning gives rise to a substantial increase in aerogel surface area and porosity. Specifically, the pre-conditioned aerogel (rGO_{30s}) exhibits a 9 times larger specific surface area (634 $m^2\cdot g^{-1}$) compared to the parent GO aerogel (68 $m^2\cdot g^{-1}$). Similarly, substantially increases are observed in aerogel porosity upon pre-conditioning, with aerogel mesopore volume increasing from 0.34 $cm^3\cdot g^{-1}$ to 1.93 $cm^3\cdot g^{-1}$ while aerogel micropore volume increases by more than 10-fold. These substantial changes further confirm that brief high-temperature Joule-heating conditioning is highly effective in removing organic impurities remaining from the aerogel synthesis and in exposing existing micro- and meso-pores within the aerogel network. The pre-conditioned aerogels also exhibit markedly changed wettability. Water droplet contact angle measurements of the parent GO aerogels indicate highly hydrophilic character (contact angle 0°), while the pre-conditioned aerogels exhibit clear hydrophobic character (contact angle 143°), confirming removal of polar oxygen surface groups and synthetic impurities after relatively short (30 s) high-temperature Joule-heating.

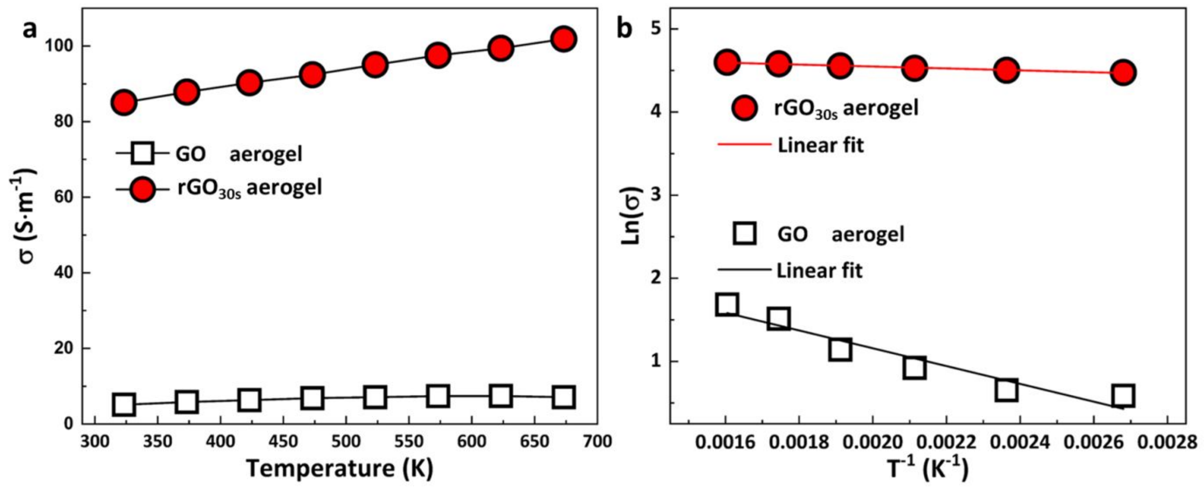

Figure S5. (a) Electrical conductivity as function of Joule-heating temperature of GO aerogel and GO_{30s} aerogel. (b) Arrhenius model fitting of GO aerogel and rGO_{30s} aerogel (bandgap for GO aerogel is 0.09 eV and for rGO_{30s} aerogel is 0.01 eV).

High-temperature Joule-heating also substantially changes the electronic aerogel properties. This change is reflected by a substantial increase in electronic conductivity from 1.6 $S{\cdot}cm^{-1}$ (for the partially reduced as-synthesised GO aerogel) to 81.5 $S{\cdot}cm^{-1}$ (after 30 s high-temperature Joule heating). Joule-heating enables a straightforward route to measure the temperature dependence of the electrical conductivity across a wide temperature range. The Arrhenius thermal activation model can therefore be readily applied to estimate the band gap of the aerogels before and after Joule-heating:[1]

$$\sigma(\mathrm{T}) = \sigma_a exp\left(-\frac{E_a}{k_B T}\right) \qquad \text{Equation (1)}$$

where σ_a is a pre-exponential factor; E_a is the activation energy; and k_B is the Boltzmann's constant. To estimate the band gaps, the logarithm of the electrical aerogel conductivity is plotted against the inverse temperature. Taking the activation energy E_a of the Arrhenius fit as estimate for the bandgap, the band gap substantially decreases from 0.09 eV (GO aerogel) to 0.01 eV (rGO_{30s} aerogel) upon 30 s Joule-heating annealing, in line with the changes in graphitic crystallinity, discussed in the main text. It is also worth noting that the fit is considerably better for the ultrahigh-temperature Joule-heated sample, highlighting again the structural stability of the aerogels after the high-temperature conditions explored in this study.

Joule-heating annealing of GO aerogels

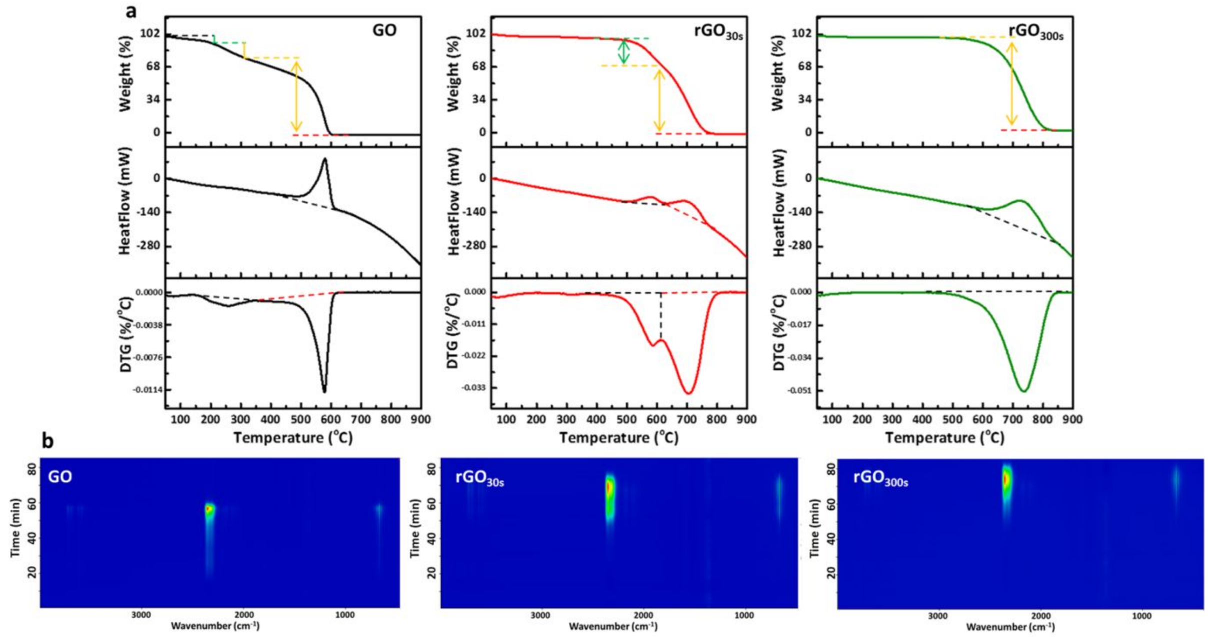

Figure S6. (a) TGA-derived analyses of GO aerogel, rGO_{30s} aerogel and rGO_{300s} aerogel. (b) TGA-FTIR 2D mapping of GO aerogel, rGO_{30s} aerogel and rGO_{300s} aerogel.

As outlined in the main text and above, short-duration, high-power Joule-heating treatments of GO_{HT} aerogels (120W power input) result in substantial GO graphitisation and annealing, as confirmed by XRD, Raman and TEM (main-text Figure 1). The evolution of graphitic quality is further reflected in substantially improved combustion resistance after Joule-annealing as probed by TGA (Figure S6a). For the parent GO aerogels, the differential thermogram (DTG) exhibits two distinct combustion events at 250 °C and 576 °C. The event at 250 °C indicates impurity removal and GO deoxygenation, while the event at around 580 °C is likely due to combustion of the (partially reduced) GO. For the rGO_{30s} sample, the lower-temperature event is no longer observed, confirming complete deoxygenation and impurity removal after only 30 s Joule-heating. In addition, the DTG of rGO_{30s} aerogel (Figure S6a) also suggests substantial annealing of the graphene basal planes as indicated by the reduced amplitude of the 585 °C event (partially-reduced GO) and the appearance of a new combustion event at considerably higher temperatures (705 °C, likely combustion of highly graphitised rGO). This effect is even more pronounced for the rGO_{300s} aerogel where only the high-temperature combustion event is observed (Figure S6a). The shift to higher combustion resistance upon Joule-heating is further evidenced through TGA-FTIR analysis, where the evolution of CO_2 combustion product (characteristic CO_2 IR peaks at ~2350 cm^{-1}, Figure S6b is observed at considerably higher temperatures for the rGO_{30s} and rGO_{300s} samples, compared to the GO_{HT} parent material.

These TGA findings are fully in line with the Raman and XRD results and further highlight the utility of high temperature Joule heating for effective enhancements of rGO aerogel quality, both in terms of graphitic crystallinity as well as aerogel purity.

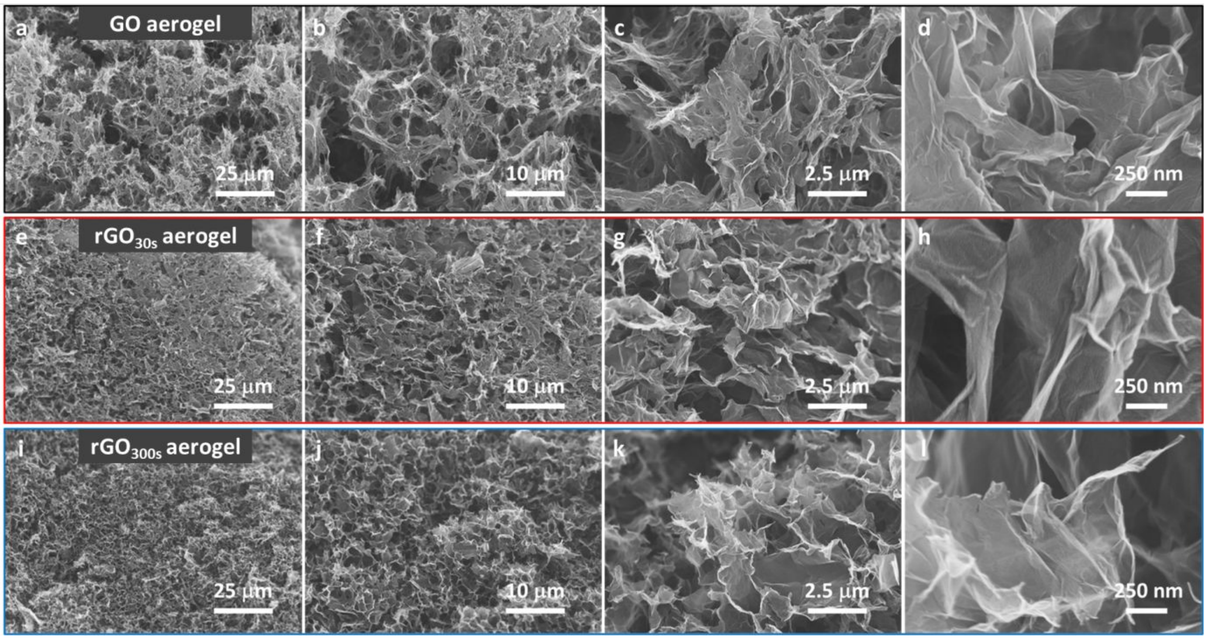

Figure S7. SEM images of GO aerogel (a-d), rGO_{30s} aerogel (e-h) and rGO_{300s} aerogel (i-l) at different magnifications.

The as-synthesised GO aerogels exhibit an internal microstructure with heterogeneous, cellular macroposity, originating from the templating by ice-crystals during the aerogel fabrication process (Figures S7a-d). SEM imaging indicates that Joule-heating annealing does not substantially alter the general macropore structure, however average macropore size slightly decreases for the 30s (Figures S7e-h) and 300s (Figures S7i-l) Joule-annealed samples. However, the pore walls seem more crumbled and porous in themselves. As evidenced by Raman and XPS data (main text, Figure 1), this is unlikely due to local combustion and is more likely due to local deformations of the multi-layer graphene stacks at the ultra-high temperatures applied. Higher magnification SEM images of the macropore walls show smooth and relatively thin rGO sheets for all samples.

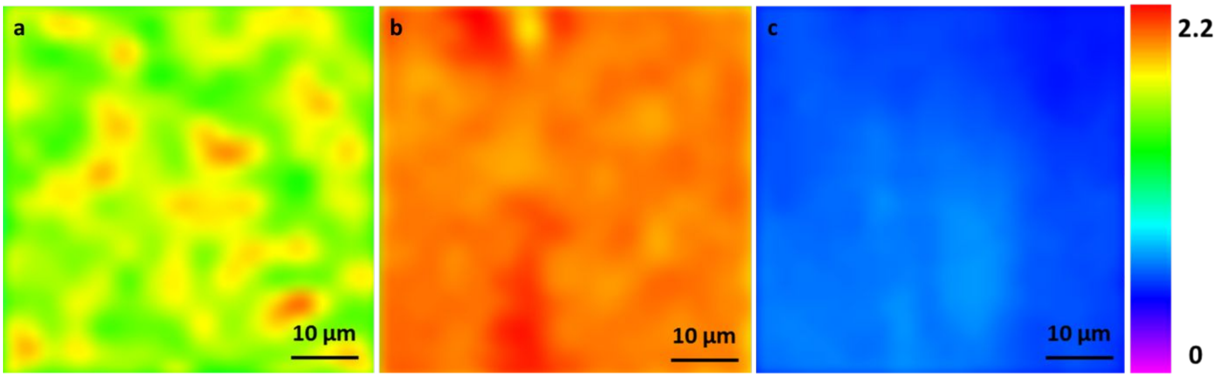

Figure S8. Raman maps, depicting variations in the intensity ratio of the D peak to the G peak (I_D/I_G) for the (a) GO aerogel, (b) rGO_{30s} aerogel and (c) rGO_{300s} aerogel across 50um x 50 um viewing field.

Improvements in graphitisation and graphitic homogeneity upon Joule-annealing are further evidenced by Raman mapping (Figure S8). The average I_D/I_G ratios in the Raman maps of the GO aerogel (I_D/I_G=1.8), rGO_{30s} aerogel (I_D/I_G=2.1) and rGO_{300s} aerogel (I_D/I_G=0.5) aerogels follow the same trend as observed by Raman spectroscopy of the bulk powders (main text Figure 1). In line with the Tungista-Konig plot for graphitic carbon materials of fundamentally different crystallinity, the substantial improvement in graphitic crystallinity for the highly disordered GO materials after 30 s high-temperature Joule heating (T_{core} ~ 3000 K) is indicated by an increase in I_D/I_G ratio (accompanied by substantial narrowing of peak width, see also Raman spectra Figure 1 main text). Further, high temperature annealing of the now relatively crystalline rGO_{30s} material is then indicated by a decrease in I_D/I_G ratio for the rGO_{300s}. As for the I_{2D}/I_G Raman maps discussed in the main text, I_D/I_G Raman maps also indicate an overall improvement of graphitic homogeneity with increasing Joule-annealing, as indicated by the more uniform colour distributions in the maps with increasing Joule-annealing duration.

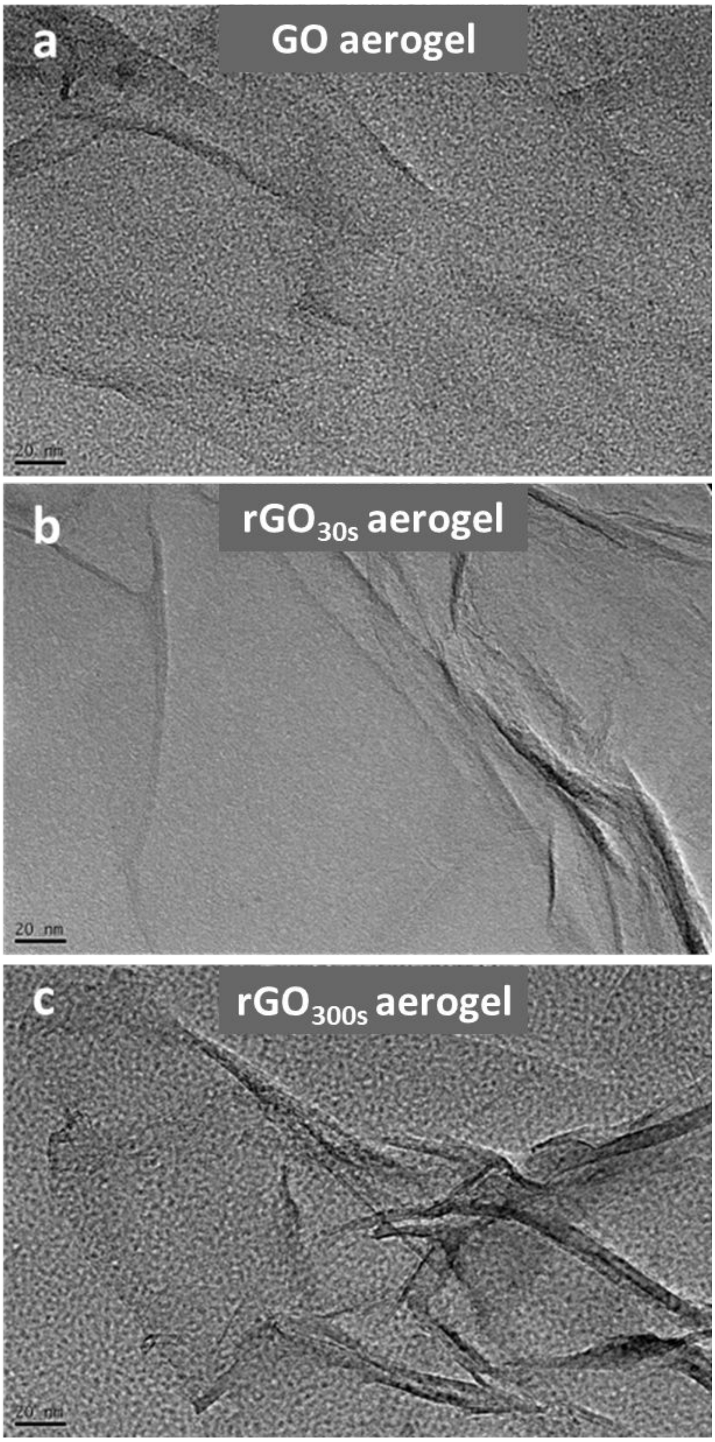

Figure S9. TEM images the (a) GO aerogel, (b) rGO_{30s} aerogel and (c) rGO_{300s} aerogel

Additional TEM images for the as-synthesised and Joule-annealed aerogel samples are presented in Figure S9. In contrast to the images discussed in the main text (Figure 1), these images focus on flatter areas in the samples. These images highlight that aerogels exhibit very thin nanosheets, demonstrating the good exfoliation of GO starting materials and suggesting limited and relatively uniform graphene sheet re-stacking upon Joule-annealing. As for the

TEM images discussed in the main text, the rGO_{300s} sample shows the clearest indication of re-stacking on the nanoscale, as indicated by more pronounced edge-features in the corresponding TEM image (Figure S9c).

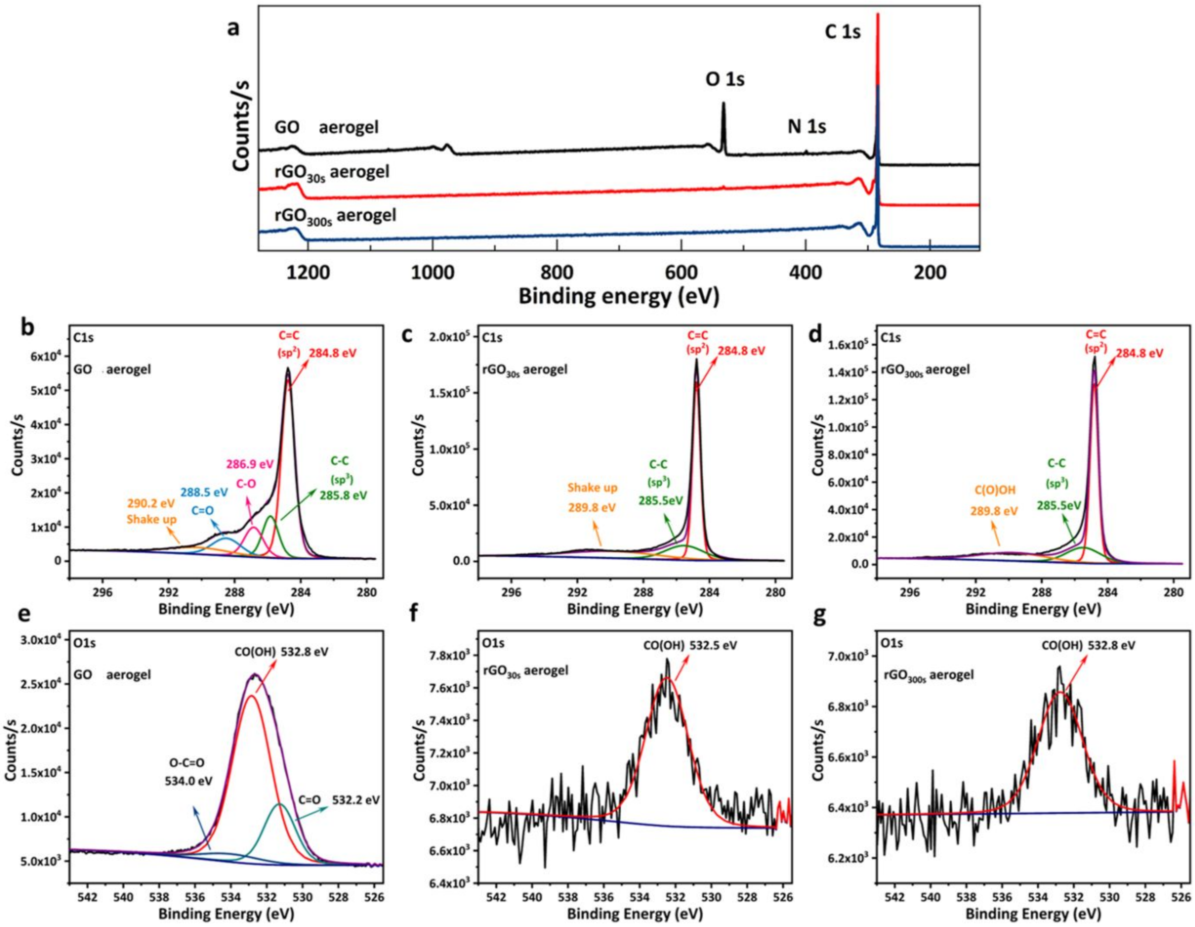

Figure S10. (a) XPS overview spectra, (b-d) XPS C1s high resolution spectra and, (e-g) XPS O1s high-resolution spectra of the (a) GO aerogel, (b) rGO_{30s} aerogel and (c) rGO_{300s} aerogel, corresponding to the XPS data, presented in the main-text Fig 3 and main-text Table 1.

Estimation of Joule-Heating Temperatures for Monolithic Aerogels

To determine aerogel temperature as function of electrical power input, rGO_{30s} aerogels were Joule-heated in inert atmosphere across a power input range of 0.5 – 120 W. The Joule heating temperature of three-dimensional, uninsulated aerogel monoliths will be higher at the core compared to the aerogel surface due to heat conduction and thermal losses at the aerogel surface. Therefore, for aerogels, both core temperatures and surface temperatures need to be considered. To this end, aerogel temperatures were estimated via a previously published model, based on simplified one-dimensional heat conduction, that links aerogel surface temperature (T_{surf}) to aerogel core temperature (T_{core}) at a fixed electrical power input (q) via the aerogel's thermal conductivity (k).[2]

$$T_{core} = T_{surf} + \frac{q}{4k}r^2 \qquad \text{Equation (2)}$$

Due to technical limitations of the temperature measurement instruments (thermocouples, high-temperature thermal cameras), aerogel Joule-heating temperatures were estimated via slightly different approaches within the medium-temperature and high-temperature regimes, as outlined in the following sections.

Medium-Temperature Regime: Aerogel Core Temperature ≤ 1200°C

In the medium temperature regime (rt – 1200 °C), regular thermocouples can be readily used to measure both T_{core} and T_{surf}. Specifically, a thermocouple was inserted into the centre of the rGO_{30s} aerogel, while a second thermocouple was brought into contact with the aerogel surface (distance, r, between core and surface position ca 5 mm, Figure S11). T_{core} and T_{surf} were then recorded at different power inputs between 0.5W to 60 W (Figure S12a). For each power input step, the measured T_{core} and $T_{surf,}$ were also used to estimate aerogel thermal conductivity k via Eq (1). Figure S12b and Figure S12c show the obtained thermal conductivity as function of Joule heating surface temperature and as function of Joule-heating core temperature, respectively. Both relationships show a typical Umklapp scattering profile with a clear inflection point, where an initial increase of k with temperature switches over to a clear decrease of k with temperature, typical for graphitic materials.

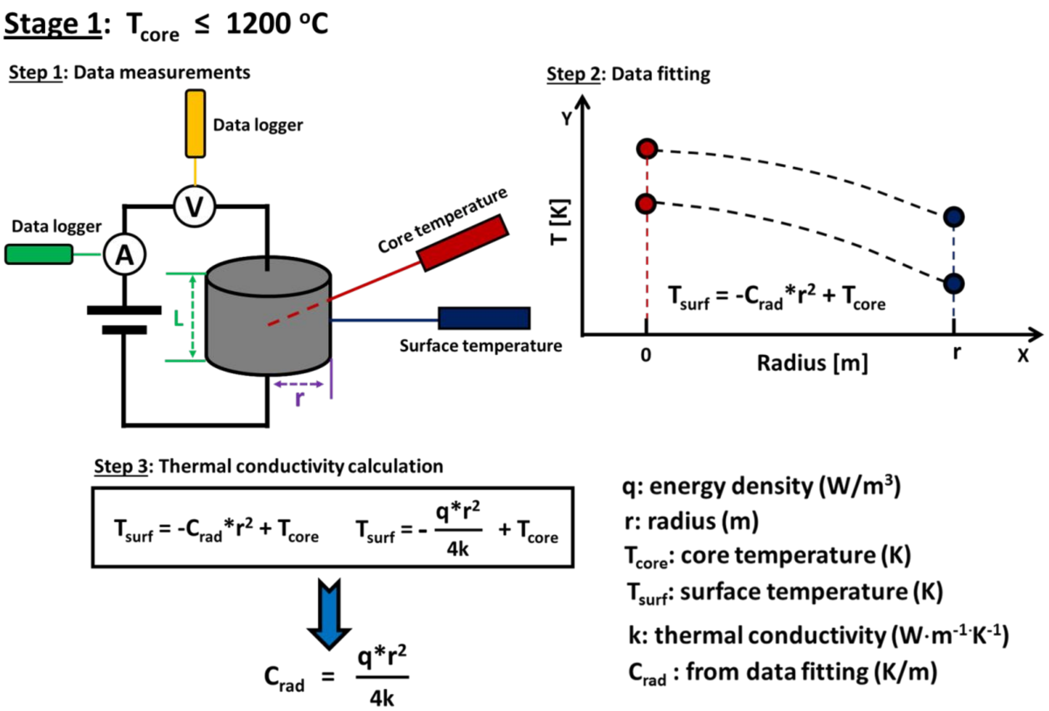

Figure S11. Schematics of estimating thermal conductivity of a rGO_{30s} aerogel (for Joule heating core temperature < 1200 ^{o}C) using a previously reported thermal gradient fitting method.

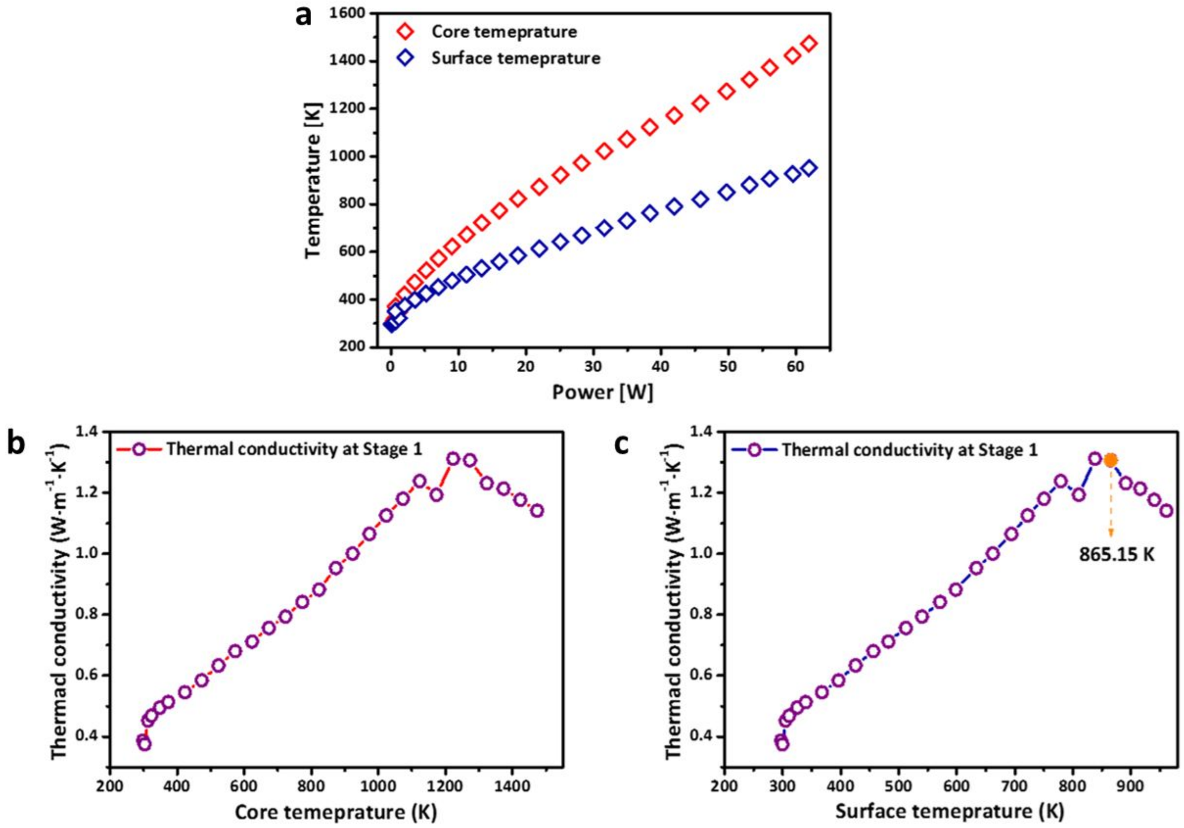

Figure S12. (a) Joule-heating core temperature and surface temperature of rGO_{30s} aerogel as function of power input. (b) Thermal conductivity of an rGO_{30s} aerogel versus Joule-heating surface temperature; (c) Thermal conductivity of an rGO_{30s} aerogel versus Joule-heating core temperature.

High-Temperature Regime: Aerogel Core Temperature >1200 °C

Thermocouples could only be reliably used for temperature measurements in the aerogel interior up to 1200 °C. Therefore, for power inputs > 60 W, aerogel core temperatures could no longer be measured directly, but needed to be estimated through equation (2) from measurements of the aerogel surface temperature. Surface temperatures could be directly measured even at very high temperatures (Figure S13), either via thermocouples (T_{surf} <1200 °C) or thermal camera (T_{surf}> 1200 °C).

However, to apply equation (2), knowledge of the thermal conductivity value κ at different surface temperatures is required. The temperature-dependence of the aerogels thermal conductivity therefore needs to be estimated. To this end, the decreasing branch of the κ-T_{surf} functional relationship in Figure S14a was extended to higher temperatures, using a previously established power-law fitting (PLF) method:[1]

$$k_{HighTemp} = k_{KnownT}\left(\frac{T_{HighTemp}}{T_{Known}}\right)^{n} \qquad \text{Equation (3)}$$

As known reference points in this equation (k_{KnownT}, T_{Known}), the values at the inflection point of the measured k-T_{surf} relationship (Figure S14a) where used, specifically k_{KnownT}=1.3 W·m^{-1}·K^{-1} at T_{Known}=865K.

$$k_{HighTemp} = 1.3\ W{\cdot}m{\cdot}K^{-1}\left(\frac{T_{HighTemp}}{865K}\right)^{n} \qquad \text{Equation (4)}$$

Where $k_{HighTemp}$ is the thermal conductivity at a measured high surface temperature $T_{HighTemp}$. The best fit of Equation (4) to the measured T_{surf} values (for all T_{surf}> 865 K) is obtained for an exponent of n=-1.31. However, the PLF model requires exponents to be integer. Therefore, fits against n=-1 and n=-2 where also carried out, with the best integer exponent fit obtained for n=-1. Taking this exponent into account, thermal conductivities can be estimated for all high surface temperatures measured.

$$k = 1.3\ W{\cdot}m{\cdot}K^{-1}\left(\frac{T_{surf}}{865K}\right)^{-1} \qquad \text{Equation (5)}$$

The high-temperature k values estimated in this way were then used as input parameters in Equation (5) in order to estimate core temperatures from the measured surface temperatures (Figure S14b). The corresponding results are depicted in Figure 2c of the main text.

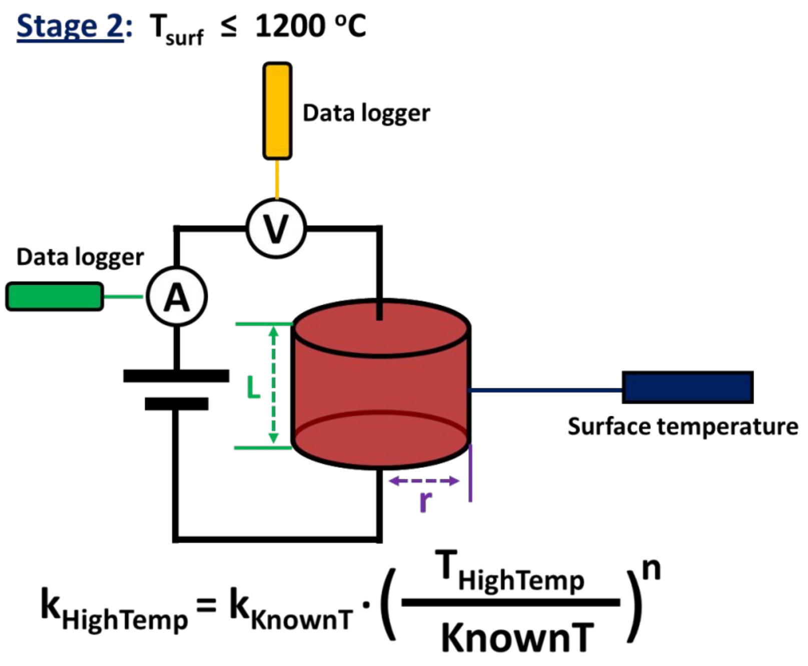

Figure S13. Schematics of estimating thermal conductivity and core temperature of an rGO_{30s} aerogel (Joule-heating core temperature > 1200 °C) using a power-law expression.

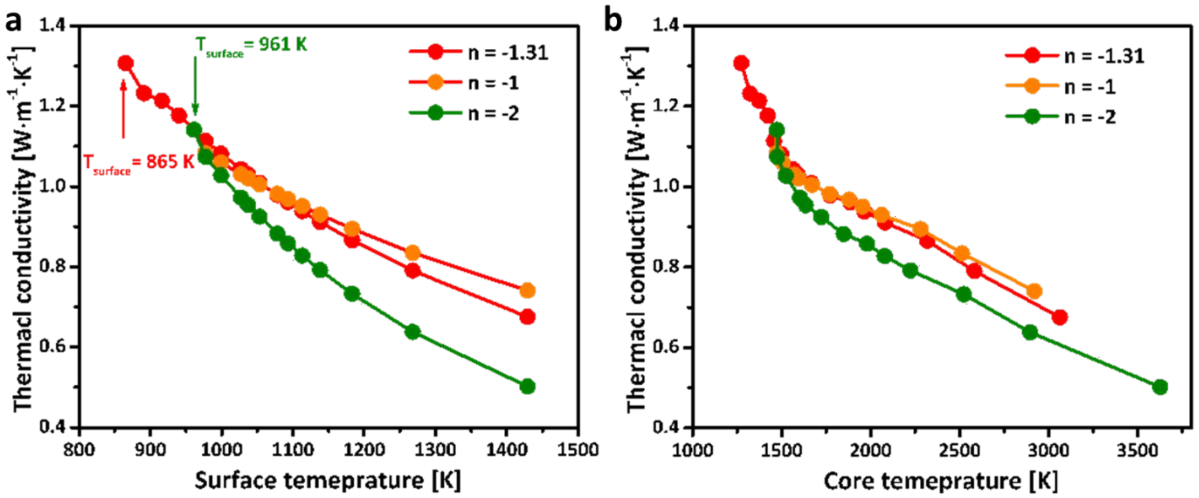

Figure S14. Thermal conductivity versus (a) Joule heating surface temperature and (b) core temperature, fitted according to Equation (3) using different n values.

Flash Joule-heating for thermo-chemical nanoparticle synthesis

PtNP@rGO aerogels

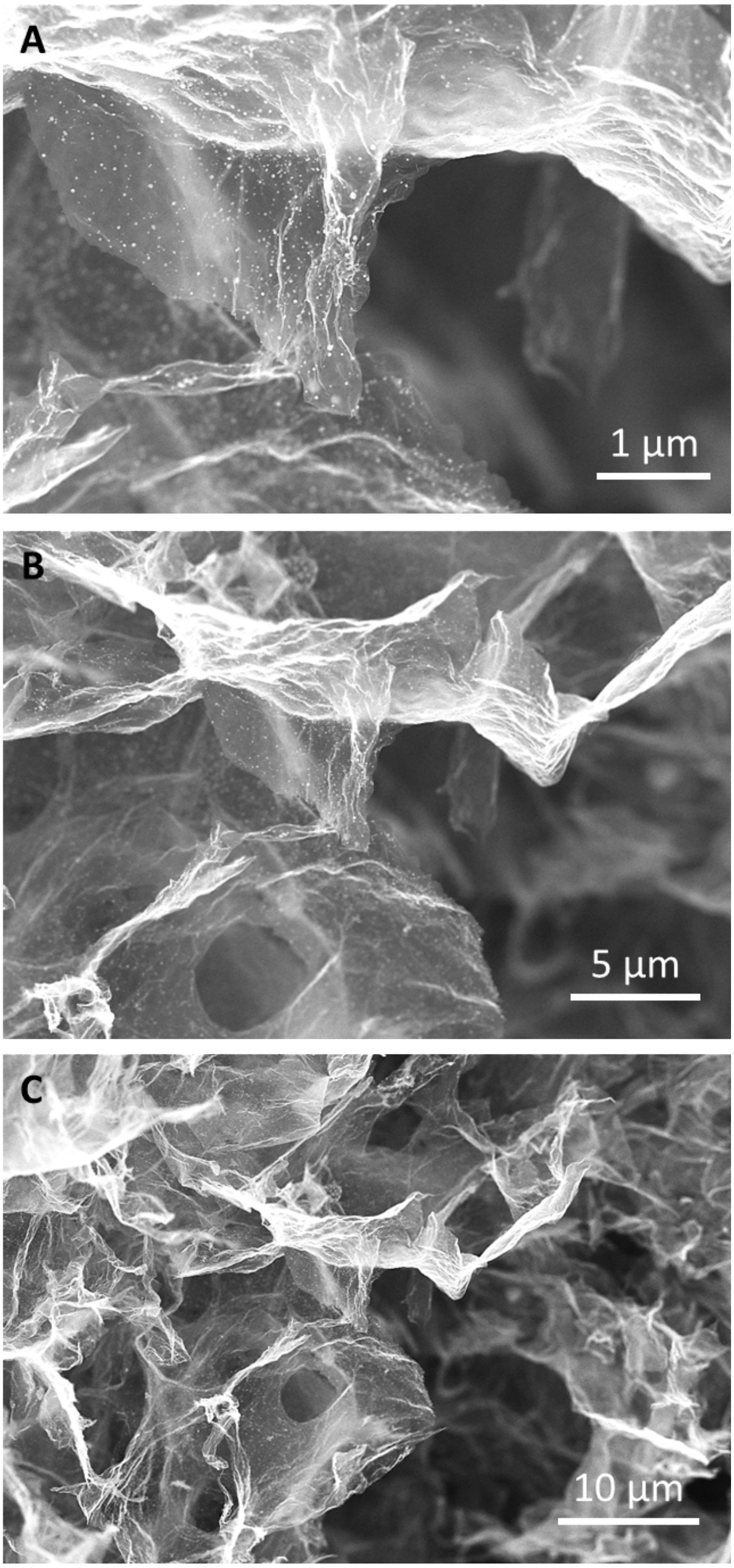

Figure S15. SEM images of Pt NP@rGO aerogels (10s flash Joule-heating).

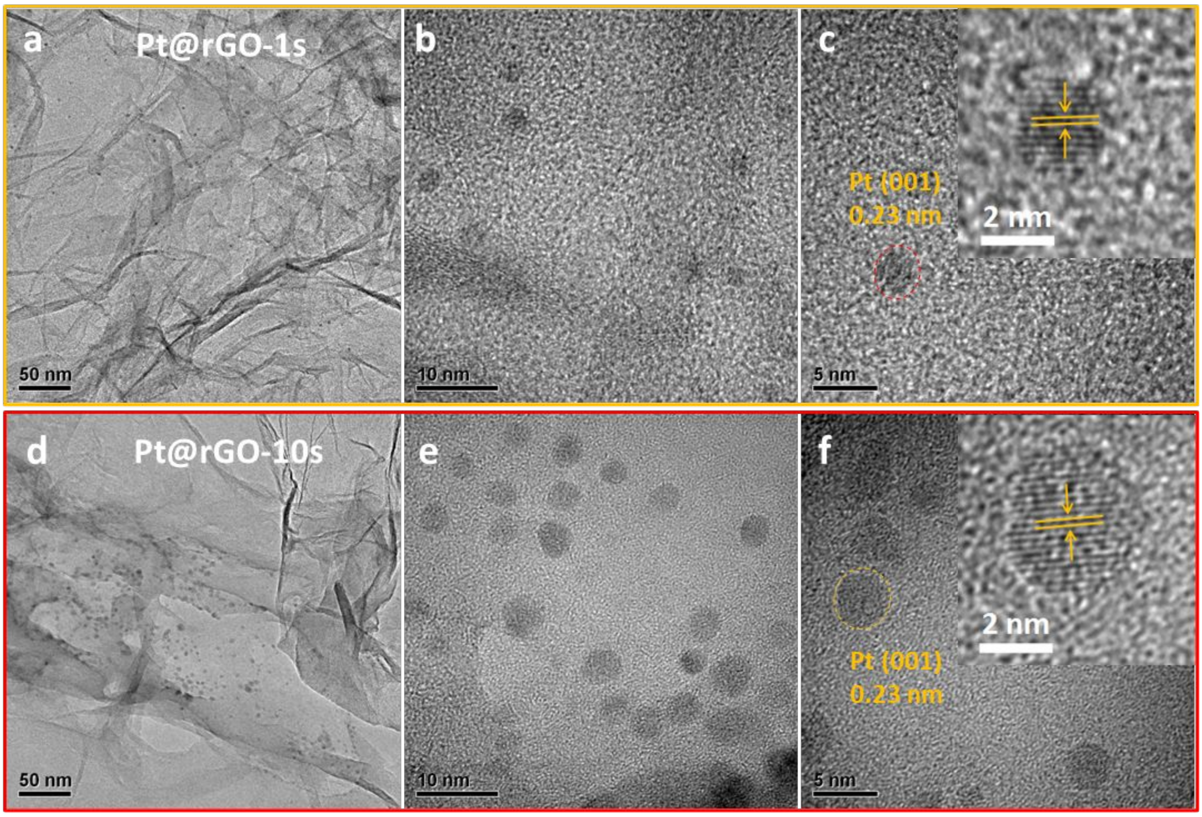

Figure S16. High-resolution TEM images for 1-second (a-c) and 10-second (d-f) flash Joule-heated Pt NP@rGO aerogel.

PtNP@rGO aerogels, produced via Pt(acac)$_2$ precursor impregnation and subsequent flash Joule heating, were characterised via XPS, XRD and Raman spectroscopy (Figure S17). Due to the low Pt weight loading and the small nanoparticle sizes, XRD does not show clear indications of the formed Pt NPs (Figure S17f). However, SEM and TEM images (discussed in the main text) clearly show the formation of small uniform nanoparticles across the aerogel network. High-resolution TEM images clearly show lattice planes, consistent with a Pt(001) lattice spacing of 0.23 nm, confirming the formation of fully crystalline, metallic Pt NPs upon flash Joule-heating. XPS also clearly indicates the presence of Pt in the functionalised samples (Figure S17a). Deconvolution analysis of the high-resolution XPS Pt 4f peak shows that Pt is predominantly present as Pt(0), in line with the formation of metallic Pt NPs, with only a minor fraction of Pt present as Pt(II), likely due to unconverted Pt(acac)$_2$ precursor (Figure S17d-e). XPS, XRD and Raman also clearly indicate that, despite the ultrahigh conversion temperature reached (T_{core} ~ 3000 K), there is no indication of combustion or oxidation of the graphitic support framework. In fact, characterisation data indicate a slight increase in graphitic crystallinity upon Joule-heating induced NP formation, in line with the Joule-heating results discussed above. For example, the C 1s high resolution XPS indicates that the fraction of sp^2

carbon very slightly increases from 49 % in 1s-flash-heated rGO aerogel (Figure S17b) to 54% in the 10-second flash Joule heated sample (Figure S17c). Similarly, the I_G/I_D ratio of the functionalised samples is significantly improved upon Joule-heating based Pt NP formation, again in line with high-temperature annealing effects. Interestingly the improvements in I_G/I_D ratio seem considerably more pronounced for the Pt-containing samples (Figure S17g), compared to the pure Joule-annealed samples discussed above and the main text. This observation suggests a potential catalytic role of the Pt-precursor compound in the annealing process, although more detailed investigations are required to confirm this observation.

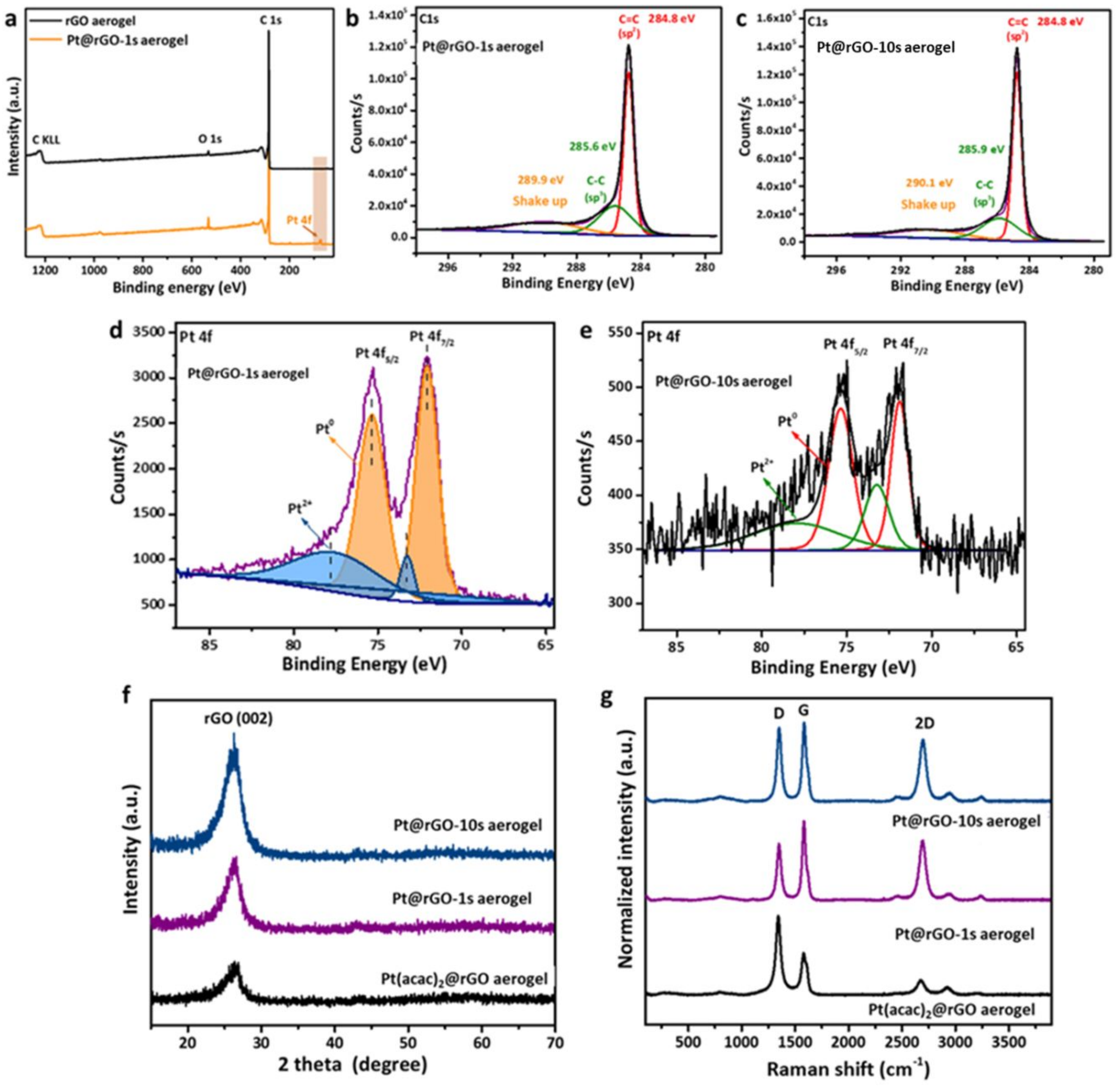

Figure S17. (a) XPS full survey spectra of rGO aerogel PtNP@rGO$_{1s}$ aerogel. (b-c), XPS C1s high resolution spectra for Pt NP@rGO aerogel flash Joule-heated for 1 s and 10 s respectively. (d-e) XPS Pt 4f high resolution spectrum for Pt NP@rGO aerogel, flash Joule-heated for 10 s. (f) XRD patterns and (g) Raman spectra for an rGO aerogel, impregnated with Pt(acac)$_2$ precursor; for a Pt NP@rGO aerogel, flash heated for 1 s; and for a PtNP@rGO aerogel, flash Joule-heated for 10 s.

CuNP@rGO aerogels

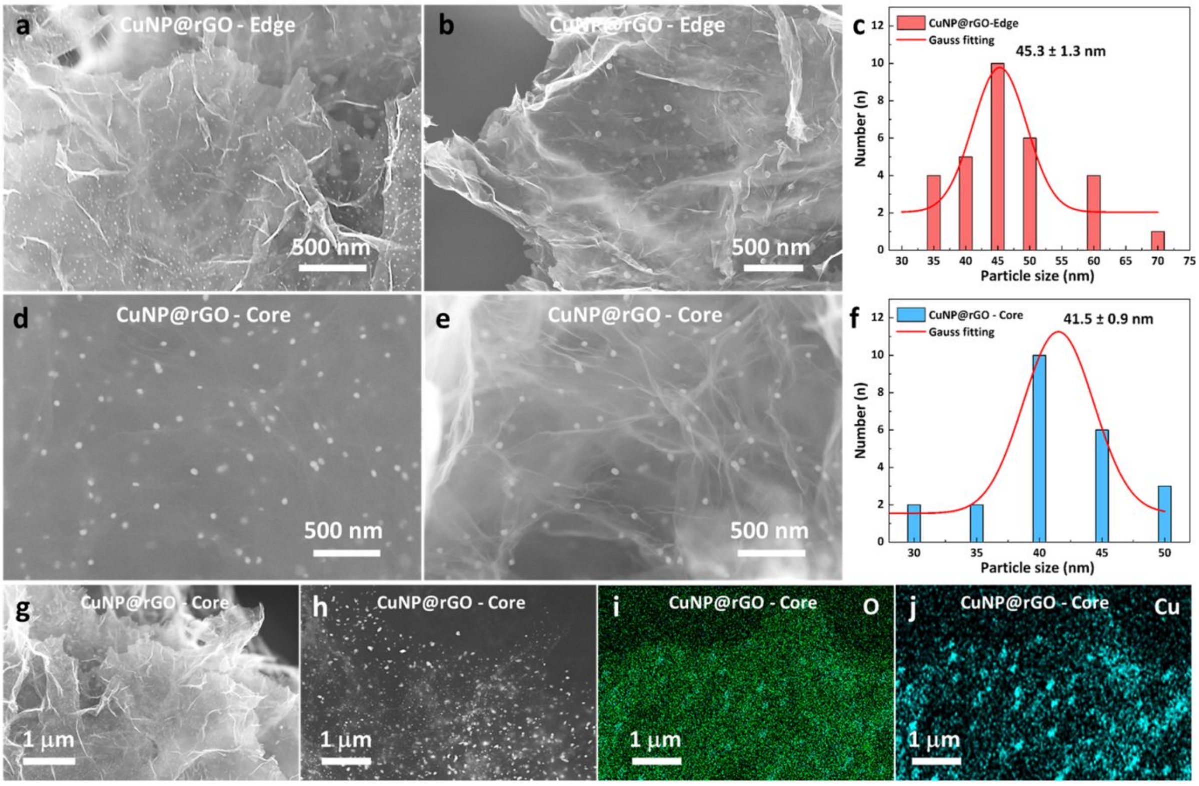

Figure S18. (a) SEM images of CuNP@rGO aerogels collected form the aerogel sample edge (a-b) and corresponding particle size analysis (c). SEM images of CuNP@rGO aerogels collected from the aerogel sample centre (d-e) and corresponding particle size analysis (f). (g-j) SEM image and EDX mapping of the CuNP@rGO aerogel collected from the aerogel sample centre.

MoO_2NP@rGO aerogels

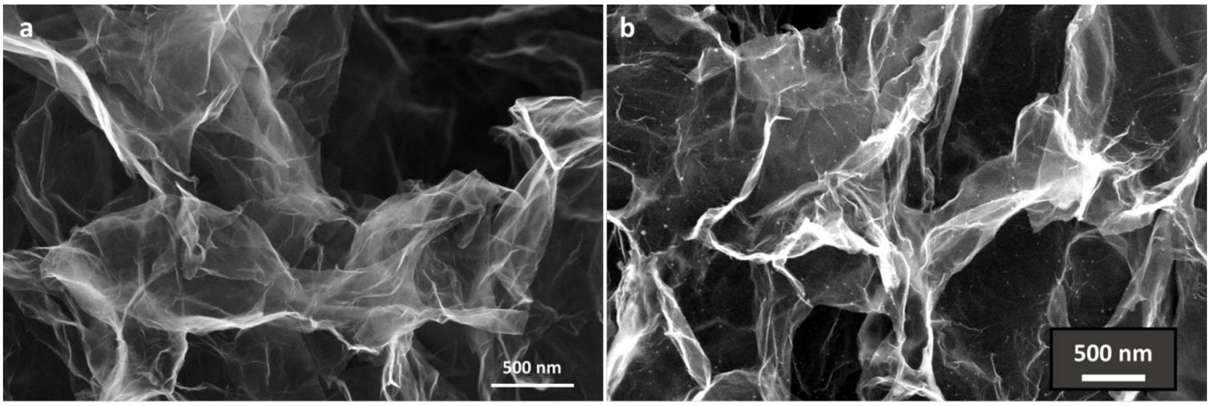

Figure S19. (a) SEM image of rGO aerogel. (b) SEM image of MoO_2NP@rGO aerogel, produced through 1s-flash Joule-heating precursor conversion.

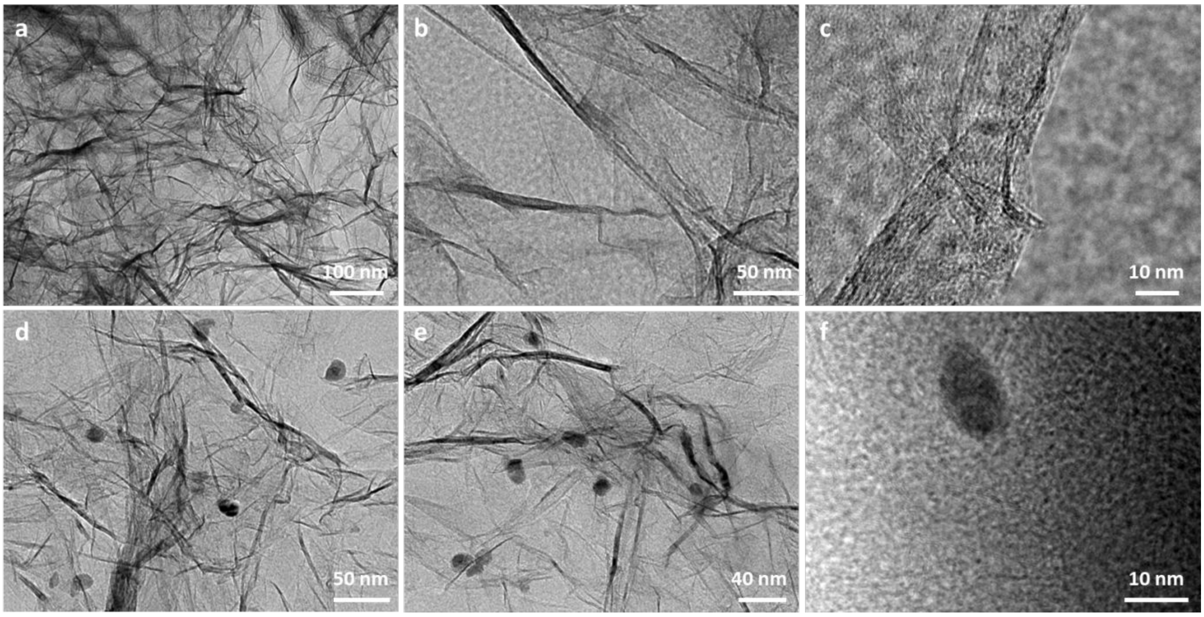

Figure S20. (a-c) TEM images of rGO_{HT} aerogel at different magnifications. (d-f) TEM images of MoO_2NP@rGO aerogel, produced through 1s-flash Joule-heating precursor conversion.

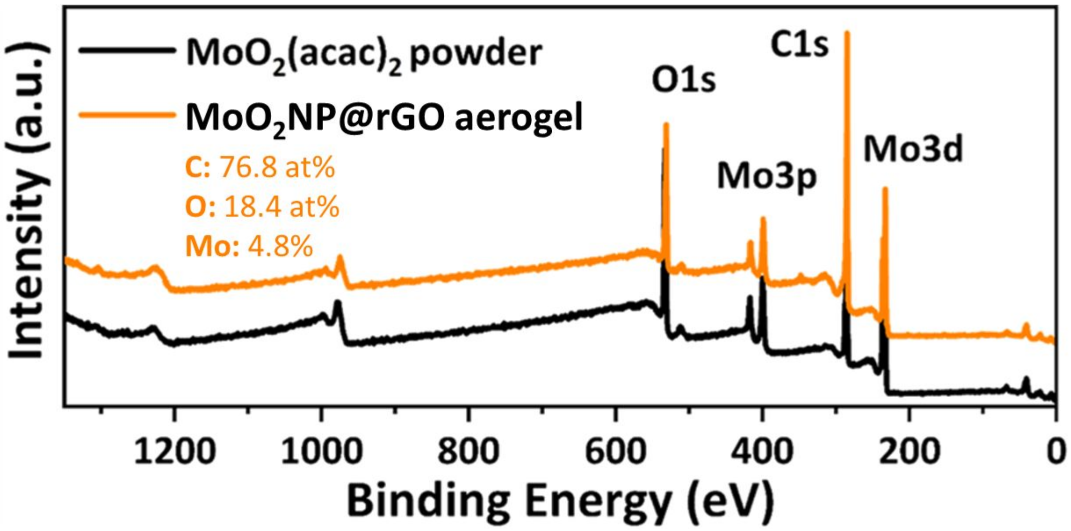

Figure S21. XPS full spectra of $MoO_2(acac)_2$ powder and MoO_2NP@rGO aerogel.

Table S2. Summary of nanocarbon-based Joule-heaters in Figure 2i.

Name	Temperature (K)	Voltage (V)	References
rGO-CNT film	343<br>355.8	5<br>6	3
Graphene-epoxy film	335<br>383<br>453	20<br>30<br>40	4
rGO fiber	1292	48.9	5
rGO sponge	623<br>673	50<br>55	6
rGO-Cu&Ni film	323<br>333<br>343	0.5<br>1<br>1.5	7
graphene molecular heater	461<br>673<br>873	3<br>4.3<br>7.5	8
Graphene glass	328<br>345<br>378	25<br>30<br>35	9
Graphene probe	1386<br>1621<br>1745<br>2749<br>2639<br>2897	28.5<br>34.8<br>41.3<br>53<br>53.2<br>59.8	10
rGO film	2460	6.3	1
Graphene film defogger	318<br>326<br>339<br>343	16<br>20<br>24<br>28	11
rGO film	1380<br>1530<br>1710<br>1780<br>2200	5.6<br>6.93<br>6.2<br>5.68<br>7.27	12
rGO film	303<br>333<br>377<br>421	1.5<br>3<br>4.5<br>6	13

	478<br>528	7.5<br>9	
Graphene aerogel	663	9	14
rGO@wood sponge	303.4<br>349<br>385.8	10<br>15<br>20	15
CNF film	1150<br>1365<br>1700<br>1950<br>2200<br>2380	3.62<br>4.61<br>5.46<br>5.82<br>6.58<br>7.87	16
CNF film	1950	15.4	17
CNF film	1150<br>1320<br>1510<br>1620<br>1760<br>1800<br>1920<br>2000	14.85<br>18.7<br>22.5<br>25.43<br>26.57<br>26.2<br>24.64<br>24.73	18
CNF film	1080<br>1150<br>1310<br>1430	32.2<br>33<br>33.85<br>32.67	19
CNT film	325<br>335<br>413<br>488	8<br>10<br>14<br>20	20
CNT film	466<br>525<br>590	6<br>8<br>10	21
hybrid CNT film	325.6<br>353.9<br>386.5	5<br>7.5<br>10	22
rGO foam	313<br>343<br>388	5<br>10<br>15	23
hybrid Graphene Membrane	315	4	24

	348<br>404<br>467<br>527	6<br>8<br>10<br>12	
rGO aerogel	323<br>348<br>393<br>453	0.5<br>0.75<br>1<br>1.25	[2]
rGO heater	1500	4	[25]
rGO aerogel	413	5	[26]
MMO/rCNT aerogel	308<br>323.5<br>346<br>370<br>399.5<br>434	1<br>1.481<br>1.933<br>2.388<br>2.833<br>3.24	[27]
rCNT aerogel	322.5<br>385<br>471.5	5.82<br>10<br>13.44	[28]
rGO aerogel	300<br>315.5<br>340<br>371.5<br>411.25<br>461.75	0.8655<br>1.6805<br>2.4365<br>3.2125<br>4.055<br>5.215	[29]
BN/rCNT aerogel	323<br>373<br>423<br>473<br>523<br>573<br>623<br>673<br>723<br>773<br>823<br>873<br>923<br>973	4.75<br>8.03<br>10.02<br>11.17<br>11.95<br>12.38<br>12.63<br>12.88<br>13.02<br>13.27<br>13.4<br>13.63<br>13.56<br>13.8	[8]

Electrothermally-driven rGO aerogel	973<br>1073<br>1173<br>1273<br>1471<br>1670<br>2063<br>2513<br>2920	6.03<br>6.41<br>6.81<br>7.22<br>8.88<br>9.67<br>10.9<br>11.3<br>11.65	This work

GO aerogel Joule-heating at shorter timescales

To probe structural evolution in GO aerogels at timescales shorter than 30s, we have produced new hydrothermal GO aerogel samples and Joule-heated these for 10s and 30s at the same conditions as outlined in the manuscript (10 A, ~120W power input). It should be noted that these additional experiments have been carried out with a different batch of commercial GO – so characterisation results for the GO aerogel and rGO_{30s} presented here differ slightly from the values presented in the manuscript's main text. Considering batch-to-batch variations in GO production, the values are however surprisingly similar and fully confirm and support the trends discussed in the main text, highlighting the robustness and broad applicability of the flash Joule-heating methodology.

In order to explore the structural evolution at shorter time scales, we characterised the GO, rGO_{10s} and rGO_{30s} aerogel samples via XPS and Raman spectroscopy. XPS elemental composition data (Figure S22, Table S3) very clearly show that after 10s Joule heating GO deoxygenation is almost complete, with a dramatic decrease of oxygen concentration from around 25at% oxygen in the parent GO aerogel to 1.5at% oxygen in the rGO_{10s} aerogel. Further Joule heating to 30s results in a small degree of additional deoxygenation (0.5 at% oxygen in rGO30s aerogel), but the data clearly indicate that 10s high temperature Joule heating are fully sufficient to induce GO de-oxygenation.

Raman spectroscopy data (Figure S23, Table S4) show that graphitisation of the lattice is also occurring on fast time scales. After 10s Joule heating, the I_D/I_G ratio reduces significantly from 1.46 to 0.57. This is accompanied by a narrowing in band widths, also indicating improvements in graphiticity, even at this short time scale. Longer Joule-heating for 30s allows for additional improvements in graphitcity. This is in line with the observations discussed in the main text

for 300s-heated sample, that showed steady improvements in graphiticity with longer heating durations.

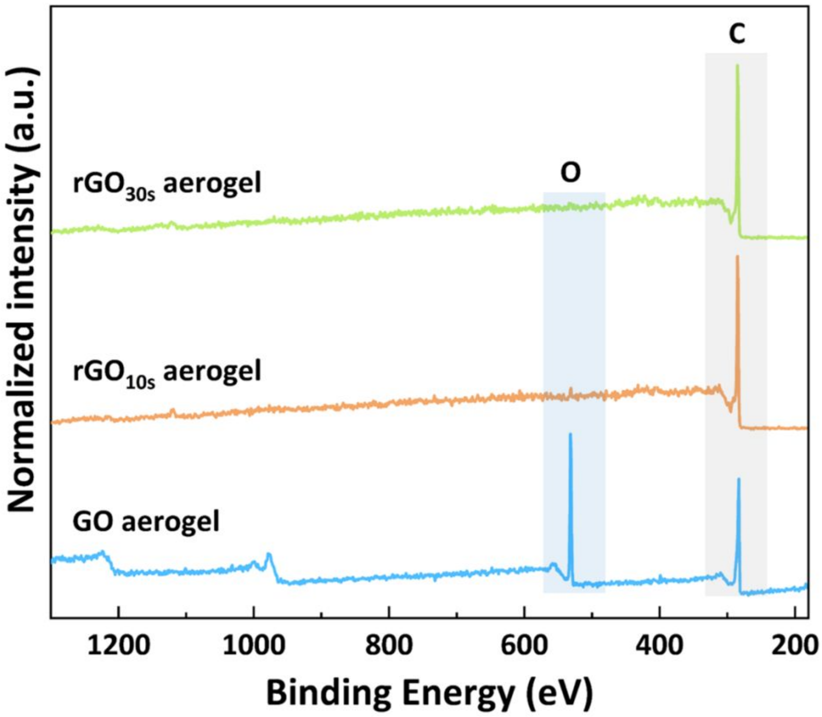

Figure 22. XPS overview spectra for hydrothermally produced GO aerogels (GO), GO aerogels Joule-heated at high current-conditions (10A, 120 W power input) for 10s (rGO_{10s}) and 30s (rGO_{30s}), respectively.

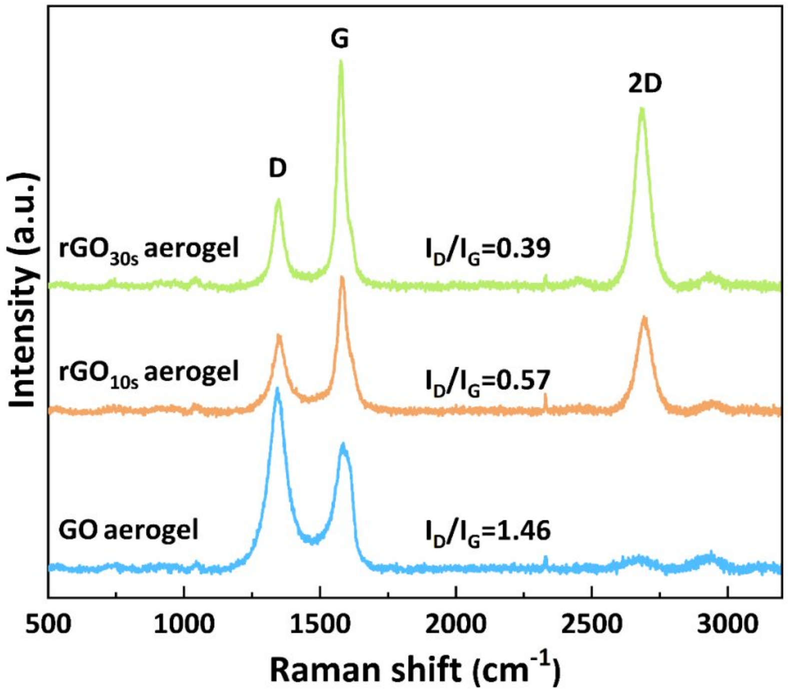

Figure 23. Raman spectra for hydrothermally produced GO aerogels (GO), Joule-heated at high current-conditions (10A, 120 W power input) for 10s (rGO_{10s}) and 30s (rGO_{30s}), respectively.

Table S3. XPS elemental composition for hydrothermally produced GO aerogels (GO), and GO aerogels Joule-heated at high-current conditions (10A, 120 W power input) for 10s (rGO_{10s}) and 30s (rGO_{30s}), respectively.

**Sample**	C (at.%)	O (at.%)
**GO aerogel**	75	25
**rGO_{10s} aerogel**	98.5	1.5
**rGO_{30s} aerogel**	99.5	0.5

Table S4. Raman spectroscopy results for hydrothermally produced GO aerogels (GO), Joule-heated at high-current conditions (10A, 120 W power input) for 10s (rGO_{10s}) and 30s (rGO_{30s}), respectively.

**Sample**	**I_D**	**I_G**	**I_D/I_G**
**GO aerogel**	4745.9	3250.1	1.46
**rGO_{10s} aerogel**	2005.9	3501.1	0.57
**rGO_{30s} aerogel**	2280.9	5900.9	0.38

Structural Stability of GO aerogels upon high-temperature Joule heating

The excellent structural robustness of our GO aerogels is surprising in the light of literature that has shown exothermic, destructive changes of GO upon medium- to high-temperature heating.[30-34] We believe that the contrast to the literature studies lies within the synthesis of the GO aerogel which was carried out under chemically reducing conditions, likely changing GO structure. Specifically, hydrothermal GO aerogel synthesis was carried out in the presence of ascorbic acid, following a well-known approach from the aerogel synthesis literature.[35] Ascorbic acid is a mild reducing agent, and has been used to aid the formation of robust GO gel networks under the mild hydrothermal synthesis temperatures used in this work. Chemical reduction by ascorbic acid likely removes some of the more labile oxygen functional groups from the GO structure, minimising violent release of gaseous products and exothermic heat upon aerogel heating in our samples (as shown for example by the DSC data in Figure S6). This chemical reduction of our GO is in clear contrast to the pristine, oxygen-rich GO, utilised in the heating studies cited above.

XRD patterns of the untreated GO and the ascorbic-acid-treated GO (i.e. the GO present in the aerogels) showed very clear differences, mainly in the (002) GO peak position and broadness (Figures S24a-24b). The untreated GO sample, showed a relatively defined XRD peak at 2theta = 11.43° (corresponding to a relatively large d-spacing of 0.77 nm), indicating the presence of a large amount of oxygen functional groups. These results are in line with literature on pristine GO, and are very similar to the XRD characteristics of the GO studied in the GO-heating papers cited above.[32-33]This is in contrast, to the ascorbic-acid treated GO (i.e. the GO present in the aerogels) used in our work that exhibited a substantial shift to smaller d-spacings (peak maximum corresponds to d = 0.36 nm), clearly evidencing chemical reduction, likely leading to partial GO de-oxygenation.

Raman spectroscopy seems to confirmed the XRD findings, showing a clear increase in I_D/I_G ratio (Figure 24c, Table S5) compared to untreated GO, suggesting some degree of sp^2 domain increase, likely associated with deoxygenation. However, the presence of ascorbic acid residues in aerogel makes Raman data interpretation uncertain. Low levels of ascorbic residues have been left in the GO aerogels on purpose in this work. (Even after four solvent exchanges around 10wt% ascorbic acid remains within the final GO aerogel structure as indicated by TGA, see also Figure S6a). Some literature studies have suggested that pyrolysis of small amounts of organics embedded in the GO network can leave behind carbonaceous residues (<1 wt%) upon heat treatment that can function as additional (covalent) crosslinking points.[36] The potential crosslinking through ascorbic acid pyrolysis is extremely challenging to evidence experimentally, due to the difficulties detecting low of carbonaceous crosslinking residues against the carbon matrix of the GO network. However, we do find that GO aerogels containing small amounts of ascorbic acid prior to heating show better structural robustness after heat treatments. This is mentioned here as potential secondary effect contributing to the stability of our aerogels under Joule-heating and to explain why we chose not to remove all ascorbic acid from the GO aerogels.

It is worth noting that literature has also shown that GO deoxygenation does occur under hydrothermal conditions,[37-38] however this effect is less pronounced at acidic conditions. The increased rGO stacking and small d(002) spacings, evidenced by XRD, do however suggest that some de-oxygenation (potentially of the more labile oxygen groups) has occurred in our materials. The large oxygen contents and large d-spacings of the pristine GO used the literature studies also suggest the presence of water in the GO interlayer spaces. Such chemisorbed

interlayer water could also significantly contribute to the disruptive structural changes in GO upon heating.[31] Finally, it should be noted that our GO aerogels are highly porous (void volume fractions of up to 95%) and possess many relatively large, spacious pores (> 5 μm) into which gaseous compounds easily can expand into upon heating, likely also substantially minimising disruptive structural changes. This is again in contrast to previous heating studies which have been carried out on much denser GO material forms, such as GO papers[30-33] and GO clumps/films.[34]

In summary, partial chemical reduction of GO through ascorbic acid during hydrothermal synthesis is indicated by XRD. This is in clear contrast to pristine GO previously investigated in the literature under medium- to high-temperature conditions. In our materials instead, chemical reduction of GO has potentially led to the removal of more reactive groups from the GO structure, resulting in materials considerably less prone to violent exothermic release of gaseous compounds upon fast heating. Secondary effects, such as additional crosslinking due to organic residue pyrolysis and the extremely high porosity of our aerogel samples might further mitigating against structural disintegration during high-temperature GO aerogel heating.

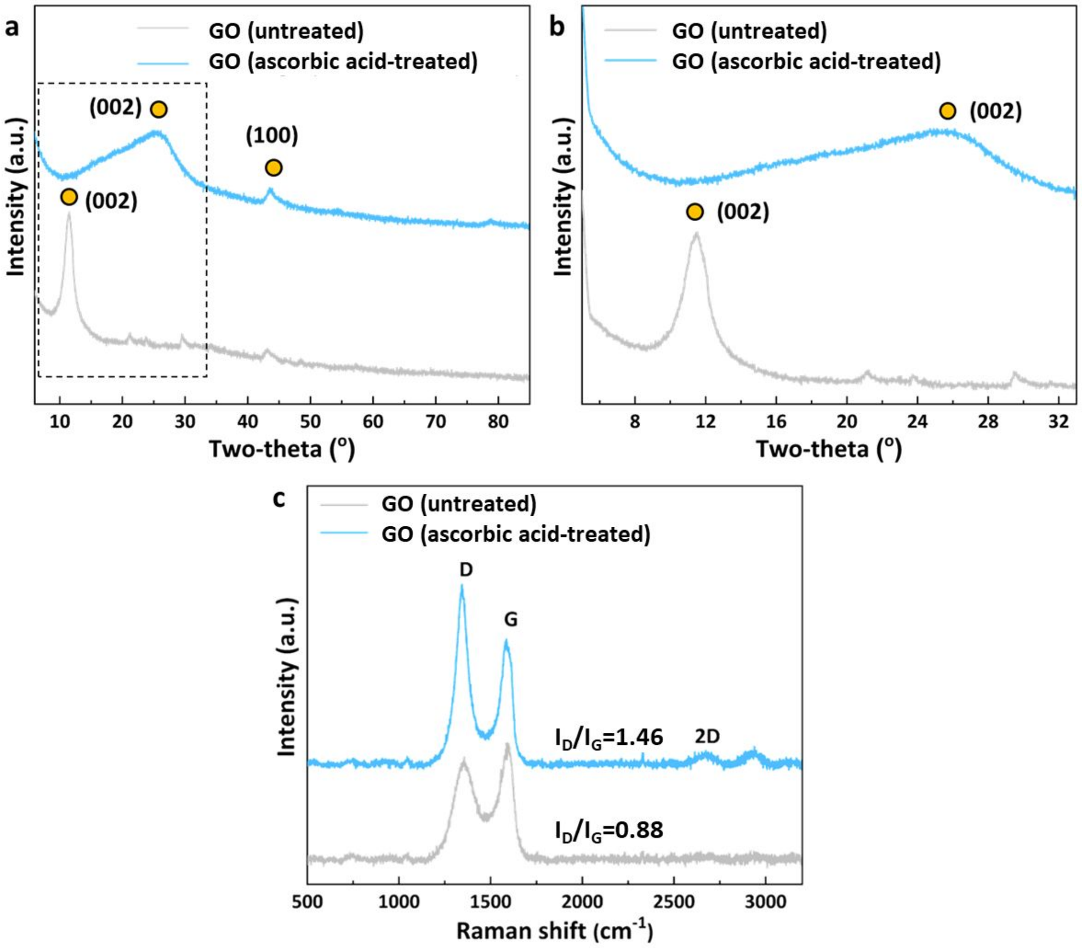

Figure 24. XRD patterns and Raman spectra of GO, hydrothermally treated with ascorbic acid (i.e. equivalent to the GO aerogel synthesis conditions), and untreated, pristine GO: (a) XRD overview patterns; (b) zoomed in XRD pattern (2theta 5-33); (c) Raman spectra.

Table S5. Raman data for and untreated, pristine GO and for GO, hydrothermally treated with ascorbic acid (i.e. equivalent to the GO aerogel synthesis conditions).

**Sample**	I_D	I_G	$I_{D/G}$
GO (untreated)	2710	3067	0.88
GO (ascorbic-acid treated)	4745	3250	1.46

Reference

1. Li, T.; Pickel, A. D.; Yao, Y.; Chen, Y.; Zeng, Y.; Lacey, S. D.; Li, Y.; Wang, Y.; Dai, J.; Wang, Y.; Yang, B.; Fuhrer, M. S.; Marconnet, A.; Dames, C.; Drew, D. H.; Hu, L., Thermoelectric properties and performance of flexible reduced graphene oxide films up to 3,000 K. *Nat. Energy* **2018,** *3* (2), 148-156.
2. Menzel, R.; Barg, S.; Miranda, M.; Anthony, D. B.; Bawaked, S. M.; Mokhtar, M.; Al-Thabaiti, S. A.; Basahel, S. N.; Saiz, E.; Shaffer, M. S. P., Joule Heating Characteristics of Emulsion-Templated Graphene Aerogels. *Adv. Funct. Mater.* **2015,** *25* (1), 28-35.
3. Zheng, Z.; Jin, J.; Xu, G.-K.; Zou, J.; Wais, U.; Beckett, A.; Heil, T.; Higgins, S.; Guan, L.; Wang, Y.; Shchukin, D., Highly Stable and Conductive Microcapsules for Enhancement of Joule Heating Performance. *ACS Nano* **2016,** *10* (4), 4695-4703.
4. Raji, A.-R. O.; Varadhachary, T.; Nan, K.; Wang, T.; Lin, J.; Ji, Y.; Genorio, B.; Zhu, Y.; Kittrell, C.; Tour, J. M., Composites of Graphene Nanoribbon Stacks and Epoxy for Joule Heating and Deicing of Surfaces. *ACS Appl. Mater. Interfaces* **2016,** *8* (5), 3551-3556.
5. Noh, S. H.; Eom, W.; Lee, W. J.; Park, H.; Ambade, S. B.; Kim, S. O.; Han, T. H., Joule heating-induced sp2-restoration in graphene fibers. *Carbon* **2019,** *142*, 230-237.
6. Ge, J.; Shi, L.-A.; Wang, Y.-C.; Zhao, H.-Y.; Yao, H.-B.; Zhu, Y.-B.; Zhang, Y.; Zhu, H.-W.; Wu, H.-A.; Yu, S.-H., Joule-heated graphene-wrapped sponge enables fast clean-up of viscous crude-oil spill. *Nat. Nanotechnol.* **2017,** *12* (5), 434-440.
7. Hazarika, A.; Deka, B. K.; Kim, D.; Jeong, H. E.; Park, Y.-B.; Park, H. W., Woven Kevlar Fiber/Polydimethylsiloxane/Reduced Graphene Oxide Composite-Based Personal Thermal Management with Freestanding Cu–Ni Core–Shell Nanowires. *Nano Lett.* **2018,** *18* (11), 6731-6739.
8. Xia, D.; Li, H.; Huang, P.; Mannering, J.; Zafar, U.; Baker, D.; Menzel, R., Boron-nitride/carbon-nanotube hybrid aerogels as multifunctional desulfurisation agents. *J. Mater. Chem. A* **2019,** *7* (41), 24027-24037.
9. Yuan, H.; Zhang, H.; Huang, K.; Cheng, Y.; Wang, K.; Cheng, S.; Li, W.; Jiang, J.; Li, J.; Tu, C.; Wang, X.; Qi, Y.; Liu, Z., Dual-Emitter Graphene Glass Fiber Fabric for Radiant Heating. *ACS Nano* **2022,** *16* (2), 2577-2584.
10. Liang, Z.; Yao, Y.; Jiang, B.; Wang, X.; Xie, H.; Jiao, M.; Liang, C.; Qiao, H.; Kline, D.; Zachariah, M. R.; Hu, L., 3D Printed Graphene-Based 3000 K Probe. *Adv. Funct. Mater.* **2021,** *31* (34), 2102994.
11. Li, Z.; Zhen, Z.; Chai, M.; Zhao, X.; Zhong, Y.; Zhu, H., Transparent Electrothermal Film Defoggers and Antiicing Coatings based on Wrinkled Graphene. *Small* **2020,** *16* (4), 1905945.
12. Chen, Y.; Egan, G. C.; Wan, J.; Zhu, S.; Jacob, R. J.; Zhou, W.; Dai, J.; Wang, Y.; Danner, V. A.; Yao, Y.; Fu, K.; Wang, Y.; Bao, W.; Li, T.; Zachariah, M. R.; Hu, L., Ultra-fast self-assembly and stabilization of reactive nanoparticles in reduced graphene oxide films. *Nat. Commun.* **2016,** *7* (1), 12332.
13. Tembei, S. A. N.; Fath El-Bab, A. M. R.; Hessein, A.; Abd El-Moneim, A., Ultrasonic doping and photo-reduction of graphene oxide films for flexible and high-performance electrothermal heaters. *FlatChem* **2020,** *24*, 100199.
14. Schütt, F.; Rasch, F.; Deka, N.; Reimers, A.; Saure, L. M.; Kaps, S.; Rank, J.; Carstensen, J.; Kumar Mishra, Y.; Misseroni, D.; Romani Vázquez, A.; Lohe, M. R.; Shaygan Nia, A.; Pugno, N. M.; Feng, X.; Adelung, R., Electrically powered repeatable air explosions using microtubular graphene assemblies. *Mater. Today* **2021,** *48*, 7-17.
15. Huang, W.; Zhang, L.; Lai, X.; Li, H.; Zeng, X., Highly hydrophobic F-rGO@wood sponge for efficient clean-up of viscous crude oil. *Chem. Eng. J.* **2020,** *386*, 123994.
16. Yao, Y.; Fu, K. K.; Zhu, S.; Dai, J.; Wang, Y.; Pastel, G.; Chen, Y.; Li, T.; Wang, C.; Li, T.; Hu, L., Carbon Welding by Ultrafast Joule Heating. *Nano Lett.* **2016,** *16* (11), 7282-7289.
17. Yao, Y.; Chen, F.; Nie, A.; Lacey, S. D.; Jacob, R. J.; Xu, S.; Huang, Z.; Fu, K.; Dai, J.; Salamanca-Riba, L.; Zachariah, M. R.; Shahbazian-Yassar, R.; Hu, L., In Situ High Temperature Synthesis of Single-Component Metallic Nanoparticles. *ACS Cent. Sci.* **2017,** *3* (4), 294-301.
18. Xie, H.; Fu, K.; Yang, C.; Yao, Y.; Rao, J.; Zhou, Y.; Liu, B.; Kirsch, D.; Hu, L., Necklace-Like Silicon Carbide and Carbon Nanocomposites Formed by Steady Joule Heating. *Small Methods* **2018,** *2* (4), 1700371.

19. Zhou, Y.; Natarajan, B.; Fan, Y.; Xie, H.; Yang, C.; Xu, S.; Yao, Y.; Jiang, F.; Zhang, Q.; Gilman, J. W.; Hu, L., Tuning the High-Temperature Wetting Behavior of Metals toward Ultrafine Nanoparticles. *Angew. Chem. Int. Ed.* **2018,** *57* (10), 2625-2629.
20. Xu, X.; Zhang, Y.; Jiang, J.; Wang, H.; Zhao, X.; Li, Q.; Lu, W., In-situ curing of glass fiber reinforced polymer composites via resistive heating of carbon nanotube films. *Compos. Sci. Technol.* **2017,** *149*, 20-27.
21. Aouraghe, M. A.; Xu, F.; Liu, X.; Qiu, Y., Flexible, quickly responsive and highly efficient E-heating carbon nanotube film. *Compos. Sci. Technol.* **2019,** *183*, 107824.
22. Hu, P.; Lyu, J.; Fu, C.; Gong, W.-b.; Liao, J.; Lu, W.; Chen, Y.; Zhang, X., Multifunctional Aramid Nanofiber/Carbon Nanotube Hybrid Aerogel Films. *ACS Nano* **2020,** *14* (1), 688-697.
23. Zeng, Q.; Ma, P.; Lai, D.; Lai, X.; Zeng, X.; Li, H., Superhydrophobic reduced graphene oxide@poly(lactic acid) foam with electrothermal effect for fast separation of viscous crude oil. *J Mater. Sci.* **2021,** *56* (19), 11266-11277.
24. Fan, T.; Su, Y.; Fan, Q.; Li, Z.; Cui, W.; Yu, M.; Ning, X.; Ramakrishna, S.; Long, Y., Robust Graphene@PPS Fibrous Membrane for Harsh Environmental Oil/Water Separation and All-Weather Cleanup of Crude Oil Spill by Joule Heat and Photothermal Effect. *ACS Appl. Mater. Interfaces* **2021,** *13* (16), 19377-19386.
25. Yao, Y.; Fu, K. K.; Yan, C.; Dai, J.; Chen, Y.; Wang, Y.; Zhang, B.; Hitz, E.; Hu, L., Three-Dimensional Printable High-Temperature and High-Rate Heaters. *ACS Nano* **2016,** *10* (5), 5272-5279.
26. Liu, Y.; Shi, Q.; Hou, C.; Zhang, Q.; Li, Y.; Wang, H., Versatile mechanically strong and highly conductive chemically converted graphene aerogels. *Carbon* **2017,** *125*, 352-359.
27. Xia, D.; Huang, P.; Li, H.; Rubio Carrero, N., Fast and efficient electrical–thermal responses of functional nanoparticle decorated nanocarbon aerogels. *Chem. Commun.* **2020,** *56* (92), 14393-14396.
28. Xia, D.; Li, H.; Huang, P., Understanding the Joule-heating behaviours of electrically-heatable carbon-nanotube aerogels. *Nanoscale Adv.* **2021,** *3* (3), 647-652.
29. Xia, D.; Xu, Y.; Mannering, J.; Ma, X.; Ismail, M. S.; Borman, D.; Baker, D. L.; Pourkashanian, M.; Menzel, R., Tuning the Electrical and Solar Thermal Heating Efficiencies of Nanocarbon Aerogels. *Chem. Mater.* **2021,** *33* (1), 392-402.
30. Compton, O. C.; Dikin, D. A.; Putz, K. W.; Brinson, L. C.; Nguyen, S. T., Electrically Conductive "Alkylated" Graphene Paper via Chemical Reduction of Amine-Functionalized Graphene Oxide Paper. *Adv. Mater.* **2010,** *22* (8), 892-896.
31. Núñez, J. D.; Benito, A. M.; Rouzière, S.; Launois, P.; Arenal, R.; Ajayan, P. M.; Maser, W. K., Graphene oxide–carbon nanotube hybrid assemblies: cooperatively strengthened OH ··· O□C hydrogen bonds and the removal of chemisorbed water. *Chem. Sci.* **2017,** *8* (7), 4987-4995.
32. Chen, H.; Müller, M. B.; Gilmore, K. J.; Wallace, G. G.; Li, D., Mechanically Strong, Electrically Conductive, and Biocompatible Graphene Paper. *Adv. Mater.* **2008,** *20* (18), 3557-3561.
33. Vallés, C.; David Núñez, J.; Benito, A. M.; Maser, W. K., Flexible conductive graphene paper obtained by direct and gentle annealing of graphene oxide paper. *Carbon* **2012,** *50* (3), 835-844.
34. Qiu, Y.; Guo, F.; Hurt, R.; Külaots, I., Explosive thermal reduction of graphene oxide-based materials: Mechanism and safety implications. *Carbon* **2014,** *72*, 215-223.
35. Liu, T.; Huang, M.; Li, X.; Wang, C.; Gui, C.-X.; Yu, Z.-Z., Highly compressible anisotropic graphene aerogels fabricated by directional freezing for efficient absorption of organic liquids. *Carbon* **2016,** *100*, 456-464.
36. Barg, S.; Perez, F. M.; Ni, N.; do Vale Pereira, P.; Maher, R. C.; Garcia-Tuñon, E.; Eslava, S.; Agnoli, S.; Mattevi, C.; Saiz, E., Mesoscale assembly of chemically modified graphene into complex cellular networks. *Nat. Commun.* **2014,** *5* (1), 4328.
37. García-Bordejé, E.; Víctor-Román, S.; Sanahuja-Parejo, O.; Benito, A. M.; Maser, W. K., Control of the microstructure and surface chemistry of graphene aerogels via pH and time manipulation by a hydrothermal method. *Nanoscale* **2018,** *10* (7), 3526-3539.
38. Li, L.; Li, B.; Zhang, J., Dopamine-mediated fabrication of ultralight graphene aerogels with low volume shrinkage. *J. Mater. Chem. A* **2016,** *4* (2), 512-518.
